# Regulation and targeting of SREBP-1 in hepatocellular carcinoma

**DOI:** 10.1007/s10555-023-10156-5

**Published:** 2023-12-01

**Authors:** Fengting Su, Andreas Koeberle

**Affiliations:** Michael Popp Institute and Center for Molecular Biosciences Innsbruck (CMBI), University of Innsbruck, 6020 Innsbruck, Austria

**Keywords:** Lipogenesis, Fatty acids, Mode of action, Cancer, Pharmacotherapy, Ferroptosis

## Abstract

Hepatocellular carcinoma (HCC) is an increasing burden on global public health and is associated with enhanced lipogenesis, fatty acid uptake, and lipid metabolic reprogramming. *De novo* lipogenesis is under the control of the transcription factor sterol regulatory element-binding protein 1 (SREBP-1) and essentially contributes to HCC progression. Here, we summarize the current knowledge on the regulation of SREBP-1 isoforms in HCC based on cellular, animal, and clinical data. Specifically, we (i) address the overarching mechanisms for regulating SREBP-1 transcription, proteolytic processing, nuclear stability, and transactivation and (ii) critically discuss their impact on HCC, taking into account (iii) insights from pharmacological approaches. Emphasis is placed on cross-talk with the phosphatidylinositol-3-kinase (PI3K)-protein kinase B (Akt)-mechanistic target of rapamycin (mTOR) axis, AMP-activated protein kinase (AMPK), protein kinase A (PKA), and other kinases that directly phosphorylate SREBP-1; transcription factors, such as liver X receptor (LXR), peroxisome proliferator-activated receptors (PPARs), proliferator-activated receptor γ co-activator 1 (PGC-1), signal transducers and activators of transcription (STATs), and Myc; epigenetic mechanisms; post-translational modifications of SREBP-1; and SREBP-1-regulatory metabolites such as oxysterols and polyunsaturated fatty acids. By carefully scrutinizing the role of SREBP-1 in HCC development, progression, metastasis, and therapy resistance, we shed light on the potential of SREBP-1-targeting strategies in HCC prevention and treatment.

## Introduction

Hepatocellular carcinoma (HCC) is the most common type of liver cancer, accounting for approximately 90% of cases, and is often accompanied by hepatitis B virus (HBV) infection or non-alcoholic steatohepatitis (NASH) as leading risk factors [1, 2]. Malignant transformation to HCC is associated with intense metabolic reprogramming, particularly related to lipid uptake, biosynthesis, (subcellular) transport, distribution, degradation, and signaling [3]. Compared to non-tumorous liver cells, which largely acquire fatty acids from extracellular sources, HCC cells develop a remarkable ability to synthesize lipids *de novo* while upregulating fatty acid uptake [4, 5]. Thus, HCC initiation and progression are highly dependent on lipogenic enzymes, such as fatty acid synthase (FASN), stearoyl-coenzyme A desaturase (SCD), acetyl-coenzyme A carboxylase 1 (ACC1), and malic enzyme (ME), and upstream regulators, including sterol regulatory element-binding protein 1 (SREBP-1), all of which are highly expressed in human HCC [4–6]. As a central lipid-anabolic transcription factor, SREBP-1 regulates a variety of (rate-limiting) enzymes in fatty acid and triglyceride biosynthesis, including ATP-citrate lyase (ACLY), ACC1/2, FASN, and SCD1 [7]. High expression of SREBP-1 and its target enzymes predicts poor survival of HCC patients and is associated with increased tumor size, high histological grade, and advanced tumor-node-metastasis stage [8, 9]. In summary, SREBP-1 plays an important role in HCC tumorigenesis and metastasis [7, 10, 11], serves as an independent prognostic marker for overall and disease-free survival of HCC patients [9], and represents a promising target for therapeutic intervention [12, 13].

## SREBP-1

SREBP-1 belongs to the SREBP family of transcription factors and contains a basic helix-loop-helix-leucine zipper (bHLH-Zip) domain for binding to sterol regulatory elements (SREs) (5′-ATCACCCCAC-3′) and E-boxes (5′-CANNTG-3′; sterol-independent SREBP interaction sites) on target gene promoters [10, 14]. SREs are also located at SREBP promoters, and autoregulatory loops have been described [15]. The three members, SPREB-1a, SREBP-1c, and SREBP-2, are encoded by two genes, SREBF1 (for SREBP-1a and c) and SREBF2 (for SREBP-2) [10]. SREBP-1a and SREBP-1c are encoded by a single gene and are transcribed from alternative start sites [16]. Accordingly, the N-terminal sequence of SREBP-1c is shorter than that of SREBP-1a, resulting in weaker (hepatic) transcriptional activity [17, 18]. Germline deletion of *Srebp-2* results in 100% lethality in mice, while the majority of homozygous Srebp-1-knockout mice do not survive *in utero*, primarily due to the loss of Srebp-1a [16]. SREBP-1a is a potent inducer of SREBP-responsive genes, regardless of whether they are involved in cholesterol, fatty acid, or triglyceride metabolism [16]. The functions of SREBP-1c are more restricted and concentrate on the expression of genes required for fatty acid biosynthesis [16]. While SREBP-2 is widely expressed [10], SREBP-1c and SREBP-1a show distinct tissue specificity: SREBP-1c is the predominant isoform in hepatocytes, and SREBP-1a is highly expressed in adipocytes and certain hepatomas [17].

**Fig. 1:**
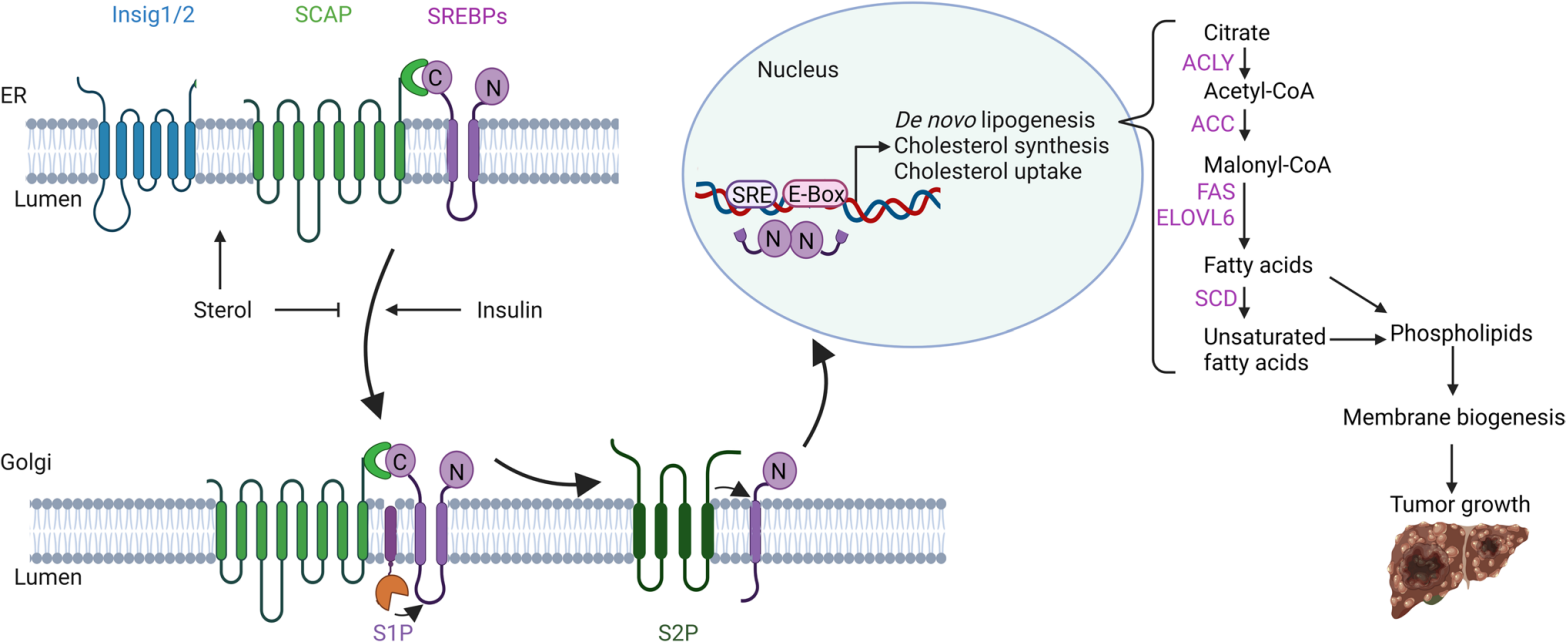
Proteolytic processing of SREBP-1 stimulates *de novo* lipogenesis and sustains tumorigenesis. pSREBP binds to SCAP and is anchored to the ER membrane by Insigs. Under low cellular sterol conditions, the SCAP-SREBP complex is released from Insigs and transferred to the Golgi, where pSREBP is cleaved by S1P and S2P, releasing the soluble mSREBP into the cytosol. mSREBP forms homodimers and translocates to the nucleus, binds to promoters with SREs or E-boxes, and transactivates the transcription of target genes. By inducing the expression of lipogenic enzymes (ACLY, ACC, FASN, elongation of long fatty acids family member 6 (ELOVL6), and SCD shown in purple) and enhancing *de novo* lipogenesis, SREBP-1 promotes membrane biogenesis, which is essential for tumor growth. Figure modified from Horton et al. and Moon et al. [16, 25] and created with https://www.biorender.com/

SREBPs are synthesized as inactive SREBP precursors (pSREBPs), which are inserted into the endoplasmic reticulum (ER) membrane and bind to the COOH-terminal region of SREBP cleavage-activating protein (SCAP) (Fig. 1) [10, 19]. The N-terminal region of SCAP functions as a sterol-sensing domain. At high cellular sterol levels, SCAP binds cholesterol, undergoes a conformational change, and in complex with SREBP interacts with insulin-induced gene 1 proteins (Insig1 and Insig2) [19]. These ER-resident membrane proteins bind cholesterol, block ER exit, and prevent the subsequent transfer to the Golgi [19]. When cellular sterol levels drop, Insig1 is ubiquitylated by the E3 ubiquitin ligase gp78 and undergoes proteasomal degradation [20, 21]. SCAP-SREBP dissociates from Insigs and is transferred to the Golgi, where SREBPs are subjected to proteolytic cleavage by site-1 protease (S1P) and subsequently S2P [22]. The mature N-terminal SREBP bHLH-Zip domain (mSREBP) is released into the cytosol, enters the nucleus as a homodimer, and binds to SRE- or E-box-containing promoters [13]. Cholesterol-derived oxysterols (e.g., 25-hydroxycholesterol) indicate excess cholesterol and mediate ER retention of the SCAP-SREBP complex by binding to Insigs [23, 24].

## Role of SREBP-1 in HCC progression

Lipids serve as energy reservoirs, structural components, and signaling mediators vital for maintaining cell growth and proliferation [11]. Research has highlighted the importance of lipid metabolism in driving cancer phenotypes [7], adapting to stress responses, including lipotoxicity [26], regulating cell survival [7, 27], and shaping the interplay of cancer cells with immunity and the tumor microenvironment [28–31], thereby revealing potential targets for anti-cancer therapy [32, 33]. This multifaceted landscape is tightly controlled by a multitude of enzymes and other factors responsible for lipid biosynthesis, lipid catabolism, and energy balance, which are subject to rigorous transcriptional and post-transcriptional regulation [11].

The transcription factor SREBP-1 serves as a central driver of lipogenesis, regulating the expression of multiple lipogenic enzymes involved in the biosynthesis of cholesterol, fatty acids, and triglycerides. It acts as a hub for myriad physiological and pathophysiological cellular processes, functioning in both transcriptional and post-transcriptional regulation [16, 34]. SREBP-1 promotes cell growth and viability, and loss of SREBP-1 signaling leads to severe lipotoxicity in glioma cells [26]. This lipotoxicity can be mitigated by the addition of monounsaturated fatty acids (MUFAs) and is considered to result from an imbalance between saturated fatty acids and MUFAs due to disturbed desaturation by SCD1 [26]. SREBP-1 is also involved in the regulation of ER stress and cell death. In osteosarcoma cells, the overexpression of SREBP-1 inhibits cell proliferation, upregulates the expression and phosphorylation of protein kinase RNA-like endoplasmic reticulum kinase (PERK), and amplifies the PERK-activated unfolded protein response (UPR), leading to ER stress-induced apoptosis and autophagy [35]. Silencing of SREBP-1 attenuates this stress response [35], whereas SREBP-1 upregulation by high glucose promotes cell growth and inhibits apoptosis and autophagy in pancreatic cancer cells [36]. Furthermore, SREBP-1 decreases ferroptosis sensitivity by inducing the SCD1-MUFA axis and modulates inflammatory reactions, such as those induced by obesity and toll-like receptor 4 (TLR4) [37–39].

The regulation of SREBP-1 is at the core of numerous metabolic diseases, such as obesity, diabetes, atherosclerosis, non-alcoholic fatty liver disease, hepatosteatosis, neurodegenerative diseases, and cancer [40–44]. The latter relies on membrane biogenesis and energy production for cell growth and proliferation (Fig. 1) and is often associated with enhanced SREBP-1 expression and/or activation [45–47]. In HCC, SREBP-1 activates a comprehensive lipogenic program [9], which includes the transcriptional upregulation of FASN, ACC, SCD, and ACLY and downregulation of medium-chain acyl-CoA dehydrogenase (ACADM)–mediated fatty acid oxidation [48]. SREBP-1 has been implicated in (i) promoting HCC proliferation and survival [13, 49–59], with high levels of SREBP-1 correlating with increased mortality in HCC patients [60]. Accordingly, the recently discovered SREBP-1 inhibitor, cinobufotalin, dose-dependently reduced both the HCC tumor volume and weight in tumor-bearing mice after 2 weeks of treatment [61]. In diethylnitrosamine (DEN)-induced HCC in C57 mice, dietary cholesterol suppressed HCC progression, which was ascribed to the inhibition of SCAP-dependent *de novo* lipogenesis [55]. Cholesterol deprivation had the opposite effect. (ii) SREBP-1 prevents cell death induction by adjusting lipid composition and content, thereby reducing the susceptibility of cell membranes to peroxidation and alleviating cellular lipotoxicity [51, 53, 62, 63]. (iii) SREBP-1 also increased tumor stemness, possibly by promoting ACLY-induced metabolic plasticity [13, 45, 64], and (iv) triggered epithelial-mesenchymal transition (EMT), migration, and metastasis, which is related to the regulation of Drosophila embryonic protein (Snail) stability and enhanced glycolytic activity [9, 13, 46, 52, 65, 66]. Thus, HCC knockdown prominently inhibited cell migration and invasion in HCC cell lines [9]. (v) SREBP-1 promotes vascular endothelial growth factor (VEGF)–induced angiogenesis [46], (vi) contributes to inflammation sensing and the generation of a tumor-promoting, pro-inflammatory microenvironment [29, 38, 39, 53, 67, 68], which may involve a feed-forward loop in which androgen and interleukin 6 (IL-6)–activated cell cycle–related kinase (CCRK) promotes non-alcoholic steatohepatitis (NASH)–derived HCC by activating the mTOR-SREBP-1 axis [69]. Blocking the SREBP pathway by liver-specific ablation of gp78 or SCAP suppressed DEN-induced hepatocarcinogenesis, resulting in reduced levels of tumor-promoting cytokines, including IL-6, IL-1β, and tumor necrosis factor-alpha (TNF-α), in mice [38]. In addition, dendritic cells exposed to HCC-derived α-fetoprotein decreased SREBP-1 expression and fatty acid biosynthesis, leading to immune suppression [70]. (vii) SREBP-1 increased HCC therapy resistance [27, 37, 44, 66, 71–74], including SCD1- or glycolytic-activated chemoresistance [37, 66], radioresistance through a combination of glucose and cardiolipin anabolism [71], and transforming growth factor beta 1 (TGF-β1)–enhanced immunoresistance [73]. Accordingly, inhibition of SREBP-1 activity by small-molecule inhibitors such as SI-1 or betulin increased the sensitivity of HCC tissue to radiofrequency ablation or sorafenib, respectively [44, 66].

The functions of SREBP-1 have been extensively reviewed, and the mechanisms behind SREBP-1 regulation, as well as its link to tumorigenesis, metabolism, metastasis, immune evasion, and therapy resistance, have been thoroughly addressed in recent reviews [11–13, 16, 28, 53, 75, 76]. While many of these articles refer to HCC and/or pre-malignant states, systematic reviews on HCC have been lacking. However, the emerging understanding of cancer heterogeneity demands an organ-, tissue-, cell type-, and context-dependent view on putative therapeutic strategies. In the following, we summarize and critically discuss the current knowledge on the regulation of SREBP-1 in HCC and highlight pharmacological approaches directed against HCC for which interference with SREBP-1 signaling has been confirmed.

## Diversity of factors controlling SREBP-1 signaling in HCC

SREBP-1 signaling is regulated in HCC by a variety of factors, including (i) endocrine hormones such as insulin [18, 77–81], glucagon [80, 82, 83], thyrotropin (TSH) [84], thyroid hormones [85, 86], glucocorticoids [87], and sex hormones (dihydrotestosterone) [88]; (ii) para- and autocrine peptide hormones such as growth factors (hepatocyte growth hormone (HGF), hepatoma-derived growth factor (HDGF), fibroblast growth factor 21 (FGF21)) [87, 89–91], cytokines (TNF-α, IL-6, IL-17A, TGFβ) [69, 92–96], and interferon-γ (IFN-γ) [73]; (iii) membrane-localized receptor ligands such as ephrin A3 [45]; (iv) lipid mediators, e.g., prostaglandin E_2_ (PGE_2_) [57]; (v) metabolites such as (oxy)sterols [23], bile acids [97], amino acids [98], and polyunsaturated fatty acids (PUFAs) [99–101]; and (vi) hepatitis viruses such as HBV and HCV [60], to name a few. Many of these factors use common signaling routes to regulate SREBP-1 signaling. For example, insulin and growth factors signal largely through RTKs, activating the phosphatidylinositol-3-kinase (PI3K)-protein kinase B (Akt)-mechanistic target of rapamycin (mTOR) axis [102], whereas the mechanistically more diverse cytokine receptors trigger different SREBP-1-regulating signaling cascades, including STAT signaling via transmembrane receptors with associated tyrosine kinases [103, 104]. Glucagon, many lipid mediators (including PGE_2_), and some cytokines/interferons activate or inhibit adenylate cyclase via G-protein-coupled 7-transmembrane receptors (GPCRs), thereby controlling PKA activation through cyclic 3′,5′-cyclic AMP (cAMP) generation, while having numerous cross-links to other pathways regulating SREBP-1 signaling [105–107]. Oxysterols, PUFAs, thyroid hormones, and bile acids bind to nuclear receptors (such as liver X receptor (LXR), farnesoid X receptor (FXR), and peroxisome proliferator-activated receptors (PPARs)) [108–110], and amino acids are an important element of nutrient sensing via mTOR [111]. Many additional endogenous factors belonging to these subgroups or sharing signaling pathways have not been explicitly reported to regulate SREBP-1 signaling in HCC, but have receptors on hepatocytes or other liver cells that regulate liver (lipid) metabolism and very likely also affect SREBP-1 signaling. Therefore, to structure the mechanisms in the control of SREBP-1 signaling, we have extracted the common pathways from the vast number of factors regulating SREBP-1 signaling in HCC and organized the following chapters accordingly.

## Common pathways regulating SREBP-1 signaling in HCC

**Fig. 2:**
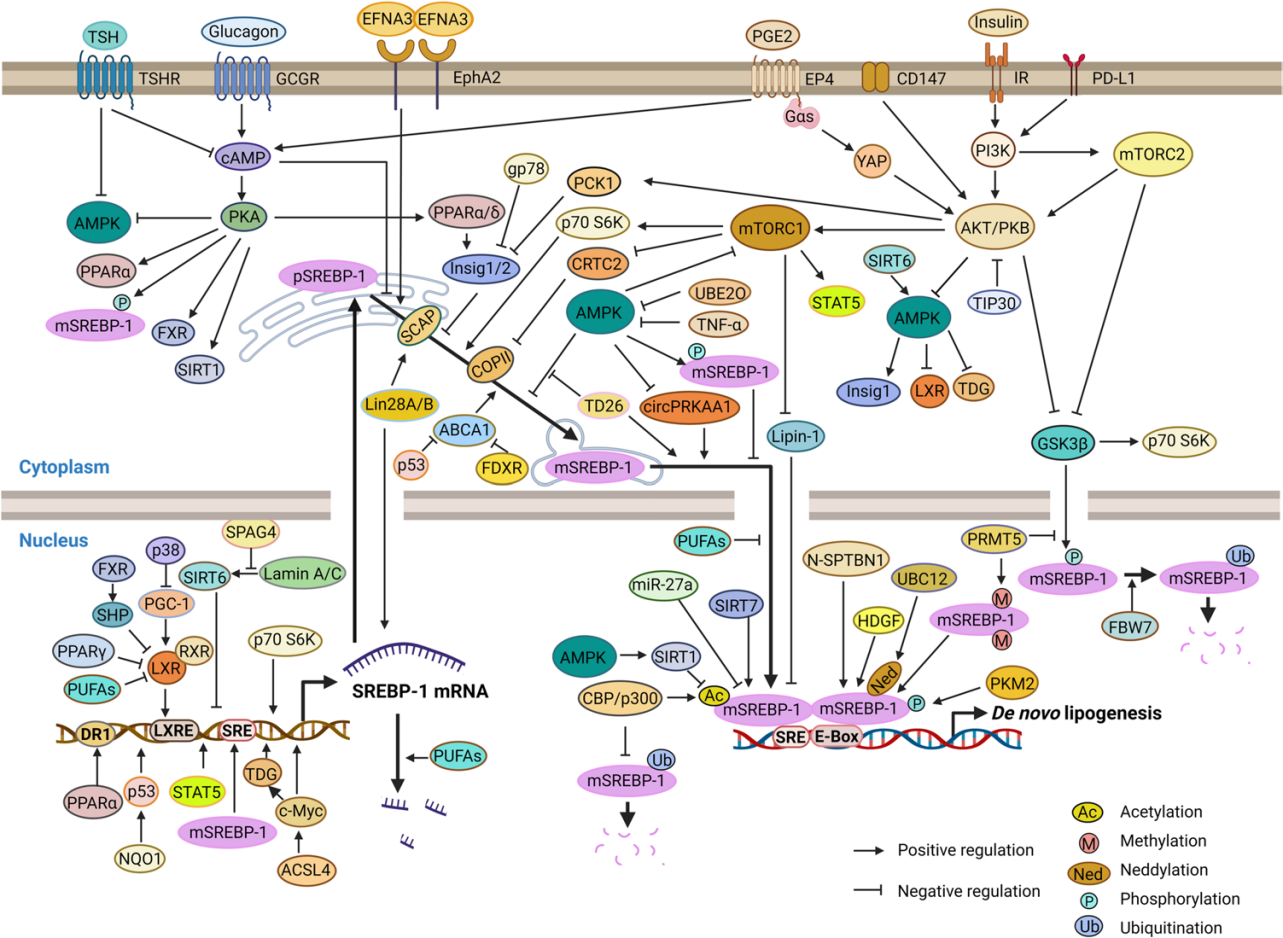
Regulation of SREBP-1 in HCC. Focus is placed on the canonical pathways that link major regulators of SREBP-1 signaling to either SREBP-1 transcription, SREBP-1 mRNA stability, pSREBP-1 maturation and intracellular trafficking, mSREBP-1 post-translational modification affecting protein stability, degradation, and transactivating activity. Major regulatory pathways that coordinate the SREBP-1 signaling include (i) the PI3K-Akt-mTOR axis, thereby considering links to lipin-1, CRTC2, TIP30, and other PI3K-Akt-regulated pathways; (ii) the serine kinases AMPK and PKA as well as other kinases that directly phosphorylate SREBP-1; (iii) the transcription factors LXR, PPARs, STAT, Myc, and p53; (iv) histone acetyltransferases, sirtuins, PRMT5, and other factors that control post-translational modification of SREBP-1; (v) metabolites, including PUFAs; and (vi) various other regulatory factors and mechanisms, such as the microRNA miR-27a, the RNA binding proteins Lin28A and Lin28B, and nuclear-localized HDGF. Created with https://www.biorender.com/

The activity of SREBP-1 is tightly regulated by a complex network of organ/tissue-specific signaling pathways at the level of (i) transcription; (ii) proteolytic processing and trafficking between the ER, Golgi, and nucleus; (iii) protein stability and degradation; and (iv) transactivating activity. It is generally accepted that SREBP-1a contributes to global lipid synthesis and growth [56, 112], whereas SREBP-1c represents a major insulin-responsive transcription factor that upregulates the expression of a variety of genes involved in (hepatic) fatty acid and triglyceride biosynthesis as well as glycolysis (i.e., glucokinase) [10, 66]. While this distinction is generally accurate, there are numerous exceptions and regulatory cross-talks, emphasizing the need for an organ/tissue/cell type–dependent understanding of the regulatory events. To provide a basis for the development of SREBP-1-targeting strategies specifically against HCC, we have compiled the current knowledge about the regulation of SREBP-1 in this cancer type (Fig. 2). We consider the tightly regulated signaling network controlling SREBP-1 transcription, maturation, subcellular localization, and transcriptional activity to be key to successful therapeutic approaches [32, 39, 50, 76]. It is now the time to address these challenges, given the recent mechanistic advances in understanding SREBP-1 regulation and signaling made possible by methodological breakthroughs in multiomics technologies, systems biology, genetic manipulation, and targeted (personalized) therapy.

Here, we concentrate on the different strategies how SREBP-1 is regulated during HCC initiation, progression, and metastasis as well as hepatic pathologies that contribute to HCC development and progression. Note that the latter studies have not explicitly investigated the impact on HCC. Furthermore, we highlight pharmacological approaches targeting liver cancer and for which a functional role of SREBP-1 interference has been confirmed or at least rationally proposed. This review is intended to help biomedical researchers working on HCC, as well as others with relevant expertise in cell metabolism, signal transduction, and lipid biology, to effectively use their interdisciplinary knowledge to address the remaining questions and find new strategies to combat aggressive (therapy-resistant) and recurrent HCC. Therefore, we comprehensively summarize the recent advances in SREBP-1 regulation; critically evaluate the conclusions drawn from the experimental data; highlight links to emerging fields, such as ferroptosis [42, 113]; and point out promising pharmacological strategies. The focus on SREBP-1, HCC, and related liver pathologies is of great importance in weighing the pros and cons of therapeutic approaches due to the substantial differences in SREBP-1 regulation between tissues, cell types, and metabolic states and distinguishes our review from others in the field. Another unique feature is that we put the spotlight on the interface between molecular signaling mechanisms, pharmacological targets, and therapeutic approaches. SREBP-1 target gene profiles and pro-tumoral signaling cascades are not the focus of this survey, and we would like to refer to excellent overview articles addressing these aspects [13, 39, 76, 114]. In addition, we would like to highlight a very recent study linking mTOR complex 1 (mTORC1)/SREBP-1c-dependent cardiolipin biosynthesis to cell survival and radioresistance in HCC [71]. Small-molecules targeting SREBP-1 signaling are listed in Table 1. Note that we only included articles that functionally linked SREBP-1 regulation/modulation to HCC or related liver pathologies and ignored others that described the effects of specific pathways or small molecules on HCC or SREBP-1 in independent studies and different systems [39, 76].

**Table 1:** Agents that have been explicitly described to inhibit SREBP-1 signaling in HCC or related liver pathologies

Target/pathway	Compound/peptide/extract	Effective concentration (*in vitro*)/dose (*in vivo*)	Reference
SREBP-1	Cinobufotalin	0.1–0.4 μM; 2.5–5 mg/kg/every other day, intraperitoneal administration (i.p.)	[61]
	SREBP decoy oligodeoxynucleotides	10 μg every 2 weeks, intravenous injection (i.v.)	[115]
SREBP-1 activation	1-(4-Bromophenyl)-3-(pyridin-3-yl)urea (SI-1)	0.26–0.88 μM; 0.5–5 mg/kg, oral administration (p.o.)	[44]
SCAP	Betulin	2.3–100 μM; 2 mg/kg/every other day, 30 mg/kg/day, intragastric administration (i.g.) or 100 mg/kg/day, i.p.	[38, 66, 116]
	Fatostatin	10–20 μM; 30 mg/kg/day, i.p.	[117]
	Lycorine	10–20 μM; 15–30 mg/kg/day, i.g.	[118]
	SCAP-siRNA lipid nanoparticles	10 nM; Single dose of 0.0625–2 mg/kg, i.v.	[119, 120]
Sec23/24	Xanthohumol	10–50 μM; 75–150 mg/kg/day, p.o.	[121]
UPS1^1^	Sulforaphane	1–100 μM; 5–20 mg/kg/day, p.o.	[122, 123]
PI3K-Akt-mTOR	Bergapten	10–50 mM; 25–50 mg/kg/day, i.p.	[58]
	Orlistat	20–50 μM; 150–300 mg/kg/day, i.p. or 10–45 mg/kg/day, p.o.	[40, 124–126]
	Tormentic acid	0.06–0.12 g/kg/day, p.o.	[127]
mTOR/CRTC2^2^	Bisphenol A	0.5–0.5 mg/kg/day, p.o.	[128]
AMPK	Metformin	0.5–20 mM 0.2–2 mg/ml in drinking water or 100–310 mg/kg/day, p.o., or 50 mg/kg/day, i.p.	[47, 79, 129–141]
	5-Aminoimidazole-4-carboxamide ribonucleotide (AICAR)	0.5–2 mM	[98, 132, 133, 135, 139, 142]
	S17834	10–25 μM; 130 mg/kg/day, p.o.	[133, 134]
	A769662	100 μM	[135]
	Antrodan	40 mg/kg/day, p.o.	[40]
	Sorafenib	0.1–50 μM; 20–30 mg/kg/day, p.o.; 0.5–2 mg/kg/every other day, i.g.; 10–30 mg/kg/every other day or 20 mg/kg/day, i.p.	[48, 98, 125, 143]
	Resveratrol	10–50 μM; 15–400 mg/kg/day, p.o.	[133, 134, 144–147]
	Apigenin	10 μM	[133]
	SY-102 (synthetic resveratrol derivative)	30–50 μM; 15–45 mg/kg/day, p.o.	[144]
	Isoquercitrin	10–200 μM; 0.5–5 mg/kg/day, p.o.	[148–150]
	Curcumin	1–10 μM; 80–100 mg/kg/day, p.o.	[151, 152]
	Astragaloside IV	50–200 μg/ml	[153, 154]
	Limonin	50–200 μM; 50 mg/kg/day, p.o.	[155]
	Nuciferine	10–20 μM; 0.10% dietary mixture, p.o.	[156]
	S-Petasin	0.5–1 μM	[157]
	S-Allyl cysteine	2–10 mM; 0.45% dietary mixture, p.o.	[158, 159]
	Betulinic acid	40 μM; 5–10 mg/kg, p.o.	[160]
	Epigallocatechin-3-gallate	0.1–1 μM; 45–125 mg/kg/day, i.p.; 31.6–100 mg/kg/day, i.g.	[161–163]
	Nordihydroguaiaretic acid (NDGA)	10–50 μM; 100–250 mg/kg/day, p.o.	[164, 165]
	MBX-2982 (GPR119 agonist)	1–10 μM; 10 μg/kg, five times weekly, p.o.	[166]
	GSK1292263 (GPR119 agonist)	0.1–3 μM	[166]
	Delphinidin-3-sambubioside	100–200 μg/ml; 15–30 mg/kg/day, p.o.	[167]
	Tormentic acid	0.06–0.12 g/kg/day, p.o.	[127]
	Ursolic acid	5–20 μM; 100–250 mg/kg/day, p.o.	[168]
	Pedunculoside	50–100 μM; 5–30 mg/kg/day, p.o.	[169]
	Fluvastatin	5 μM; 3 mg/kg/day, p.o.	[170, 171]
LXR	Resveratrol	30 mg/kg/day, p.o.	[147]
	Ursolic acid	5–20 μM; 100–250 mg/kg/day, p.o.	[168]
FXR	Schaftoside	25–50 μM; 80–160 mg/kg, p.o.	[172]
PGC-1β	Epigallocatechin-3-gallate	50 μM	[173]
PXR^3^	Phenobarbital	Single dose of 100 mg/kg, i.p.	[174]
	Pregnenolone-16α-carbonitrile	Single dose of 40 mg/kg, i.p.	[174]
	Epigallocatechin-3-gallate	300 mg/kg, i.g.	[175]
RAR^4^	AC261066, AC55649	5.4 mg/kg/day, p.o.	[176]
CAR-Insig1^5^	Phenobarbital	Single dose of 100 mg/kg, i.p.	[174]
	1,4-bis[2-(3,5-Dichloropyridyloxy)]benzene (TCPOBOP)	Single dose of 10 mg/kg, i.p.	[174]
PPARγ	Resveratrol	0.8–3.2 g/kg dietary mixture, p.o.	[177]
	Rosiglitazone	10 μM; 10 mg/kg/day, p.o.	[127, 178]
	Pedunculoside	50–100 μM; 5–30 mg/kg/day, p.o.	[169]
GPR40^6^	GW9508 (*via* AMPK activation)	50–100 μM; 100 mg/kg/day, p.o.	[179]
	AMG-1638	3 μM	[180]
	Docosahexaenoic acid	300 μM	[180]
PKM2-SREBP-1a	FPGLFDPPYAGSGAGRKKRRQRRR (P8)	8 μM	[181]
Sirtuins	Fluvastatin	5 μM; 3 mg/kg/day, p.o.	[170, 171]
	Resveratrol	10 μM; 30–400 mg/kg/day, p.o.	[67, 90, 146, 147, 182]
	Pterostilbene (a resveratrol derivative)	5–10 μM; 50 mg/kg/day, p.o.	[183, 184]
	3′-Hydroxypterostilbene (a resveratrol derivative)	1–5 μM; 50 mg/kg/day, p.o.	[184]
NEDD8-activating enzyme-E1 (Nae1)	MLN4924	0.25–3 μM; 30 mg/kg, twice daily, subcutaneous administration (s.c.) or 30 mg/kg, twice weekly, i.p.	[65, 185]
SKI-1/S1P^7^	PF-429242	10–50 μM; 2.5 mg/kg/day, p.o.	[186]
DPP-4^8^	Gemigliptin (*via* AMPK activation)	1–2 μM; 330–380 mg/kg/day, p.o	[187]
	Sitagliptin	50–1080 mg/kg/day, p.o.	[138, 188]
	Linagliptin	5–10 mg/kg/day, p.o.	[188]
NSDHL^9^	Regorafenib	2.5–10 μM; 5 mg/kg, i.g. in mice; 80–160 mg/day for 3 weeks of every 4-week cycle, p.o., in patients with advanced HCC	[73]
Unknown	NDGA	35 μM	[189]
	Pyridine co-ligand functionalized Pt(II)-complexes	5–20 μM	[190]
	Piceatannol (3,3′,4,5′-tetrahydroxy-trans-stilbene, analogue of resveratrol)	100 μM; 0.1–0.25% in high-fat diet ad libitum	[191, 192]
	Hydroxytyrosol	100 μM; 10–50 mg/kg/day, p.o.	[78, 137]
	Dimethyl 1-methyl-2-thioxoindoline-3,3-dicarboxylate (TOIDC)	14.17–20 μM; 10 mg/kg/day, i.p.	[193]
	Telmisartan	10 μM; 5.2 mg/kg/day, p.o.	[138, 194]
	Plumbagin	0.5–1 mg/kg/day, p.o.	[126]

^1^*UPS* ubiquitin proteasome system, ^2^*CRTC2* CREB-regulated transcription co-activator 2, ^3^*PXR* pregnan-X-receptor, ^4^*RAR* retinoic acid receptor, ^5^*CAR* constitutive androstane receptor, ^6^*GPR40* G-protein-coupled receptor 40, ^7^*SKI-1/S1P* subtilisin/kexin-isozyme-1/site-1 protease, ^8^*DPP-4* dipeptidyl peptidase-4 inhibitor, ^9^*NSDHL* NAD(P)-dependent steroid dehydrogenase-like

## PI3K-AKT-mTOR axis

The PI3K-Akt-mTOR axis is a central signaling cascade initiated by receptor tyrosine kinases (RTKs), including the insulin receptor (IR) and growth factor receptors, and essentially regulates fatty acid, phospholipid, and neutral lipid metabolism by activating SREBP-1 [50, 195, 196]. There are manifold other pathways that converge in the PI3K-Akt-mTOR-SREBP-1-axis, including hypoxia-initiated signaling routes [57], the surface glycoprotein cluster of differentiation 147 (CD147) [197], thyroid hormones and TSH [84–86], protein kinase (PK)D3 signaling [198], and potentially the Eph ligand ephrin A3 (EFNA3) [45]. For example, hypoxia activates the PI3K-Akt-mTOR signaling cascade and thus SREBP-1 in experimental murine HCC *via* the Hippo pathway, which in turn is stimulated *via* the PGE_2_ receptor 4 (EP4) by PGE_2_ that is released from the mesenchymal tumor environment [57]. In addition, mTORC1 forms a hub for nutrient/amino acid sensing [111], which regulates SREBP-1 expression and maturation in HCC independently of PI3K and Akt [199]. The mechanisms that translate PI3K-Akt-mTOR activation into increased SREBP-1c activity are diverse and tightly cross-regulated [80, 200, 201]. This combined signaling network drives anabolic cell metabolism with effects on cell growth, proliferation, and survival and supports tumorigenesis, in addition to orchestrating a variety of other physiological and pathophysiological cellular functions [7, 8, 11, 202]. For example, mTORC1 activation leads to the phosphorylation and subsequent inactivation of cAMP response element binding protein (CREB)–regulated transcription co-activator 2 (CRTC2) [203], thereby enabling the transport of the SREBP/SCAP complex from the ER to the Golgi in the liver, dependent on component of the coat protein complex II (COPII) [204]. Moreover, mTORC1 adjusts the nuclear availability of SREBP-1 through the phosphatidate phosphatase lipin-1 [200], co-activates SREBP-1-regulatory kinases that influence maturation and stability [80], and acts on additional factors, such as the phosphorylated signal transducers and activators of transcription 5 (STAT5) [8] and the nuclear import-regulating oxidoreductase tat-interacting protein 30 (TIP30) [56].

The lipogenic function of mTOR is associated with HCC progression. Mice with liver-specific mTORC1 activation develop spontaneous HCC, which is accompanied by increased expression of SREBP-1c and lipogenic enzymes (i.e., ACC1, FASN) as well as lipid accumulation [8]. It should be noted that the predominant mechanisms by which mTOR triggers SREBP-1c signaling are cell-type- and context-dependent and that PI3K-Akt stimulates SREBP-1-mediated lipogenesis also independently of mTOR [205]. Consistent with this finding, liver-specific deletion of tuberous sclerosis 1 (TSC1), a cochaperone that negatively regulates mTORC1 signaling [206], activates mTORC1 but shows defects in SREBP-1 maturation and fails to protect against age- and diet-dependent hepatosteatosis in mice [199, 207]. Below, we describe major signaling cascades/factors (protein kinases, lipin-1, CRTC2) that are used by HCC to translate an activation of the PI3K-Akt-mTORC1-axis into SREBP-1 signaling. In addition, we highlight selected signaling cascades (CD147, thyroid hormones, EFNA3, and TIP30) that activate the PI3K-Akt pathway, induce SREBP-1 expression or activation, and have (pre)clinical potential in the context of HCC.

### Protein kinases

#### Phosphoenolpyruvate carboxykinase 1 (PCK1)

PCK1 is one of the rate-limiting enzymes in gluconeogenesis and is involved in the regulation of SREBP-1 [208]. Akt phosphorylates PCK1 at serine-90 (S90) in human Huh-7 hepatoma cells, resulting in the translocation of the kinase to the ER, where PCK1 uses guanosine-5′-triphosphate (GTP) to phosphorylate Insig1 at S207 and Insig2 at S151 [209]. Phosphorylation of Insig1/2 lowers their binding affinity to sterols, weakens their interaction with SCAP, and allows the transfer of SREBP-1/2 from the ER to the Golgi, where proteolytic processing to the mature transcription factor takes place. The proliferation of HCC cells is in consequence induced, as is tumor growth in HCC-grafted mice [209].

#### Glycogen synthase kinase 3 (GSK3)

SREBPs contain a consensus phosphopeptide motif (CPD) that interacts with F-box and WD repeat domain-containing 7 (FBW7), the substrate recognition component of a complex of Skp1, Cul1, and F-box protein (SCF)-type ubiquitin ligase [210]. GSK3 phosphorylates the CPD, thereby recruiting FBW7 to SREBPs and stimulating ubiquitination and proteasomal degradation, as shown for diverse cancer cell lines including HCC cells [112, 211–213]. Specifically, GSK3 phosphorylates human SREBP-1 at threonine-426 (T426) and S430 [112], human SREBP-2 at S432 and S436 [112], and rat SREBP-1 at S73 [214]. Inhibition of GSK3 by Akt or the Akt-activating kinase mTORC2, therefore, stabilizes mature SREBP, enhances lipogenesis, and is associated with HCC cell proliferation and survival [6, 7, 112, 211]. In this sense, mTORC2 activates Akt by phosphorylation at S473, increases mSREBP levels, induces lipid accumulation, and promotes HCC in mice deficient in hepatic Tsc1 and phosphatase and tensin homolog (Pten), and its activity correlates with lipogenesis and HCC in patients [41]. Note that the role of GSK3 in HCC remains controversial, with several studies also suggesting GSK3 as an anti-tumoral target [215].

#### p70 S6K

Activation of PI3K-mTORC1 by insulin or growth factors stimulates SREBP-1c processing *via* p70 S6K [80]. p70 S6K then phosphorylates ribosomal protein S6 (RPS6) [216], which fosters HCC progression and increases the expression of lipogenic enzymes (FASN, ACLY, SCD1, 3-hydroxy-3-methylglutaryl-CoA reductase (HMGCR), mevalonate kinase (MVK)) and promotes lipogenesis in human HCC cell lines, human HCC specimen, and liver of transgenic mice expressing constitutively active Akt [6]. The authors propose that the activation of the RPS6 pathway disrupts the FBW7-SREBP-1 and FBW7-SREBP-2 degradation complexes [6], which have previously been shown to initiate the ubiquitination and proteasomal degradation of nuclear SREBP-1a, SREBP-1c, and SREBP-2 [112].

### Lipin-1

The phosphatidic acid phosphatase lipin-1 critically regulates lipid homeostasis, either through its enzymatic activity or by co-regulating gene transcription [217]. Active lipin-1 in the nucleus evokes nuclear eccentricity, reduces SREBP promoter activity, and decreases mSREBP abundance [200]. Phosphorylation of lipin-1 by mTORC1 prevents the nuclear entry of the phosphatase and thereby mediates the effect of mTORC1 on SREBP gene expression through undefined mechanisms, as shown for murine fibroblasts, AML12 hepatocytes, and HepG2 cells *in vitro* and in the liver of mice fed a Western diet [200]. Lipin-1 phosphorylation by mTORC1 and casein kinase I further allows recognition by the SCFβ-TRCP E3 ubiquitin ligase complex and subsequent degradation [218]. Interestingly, the lipin-1 promoter contains an SRE site, which explains why SREBP-1 activation upon sterol depletion enhances lipin-1 transcription in HCC cell lines [219]. Induction of lipin-1 expression by SREBP is synergistically enhanced by the transcription factor nuclear factor Y (NF-Y) [219].

### CRTC2

CRTC2 belongs to a family of CREB co-activators and regulates glucose and lipid metabolism in the liver and other tissues [128, 204]. As shown in the liver of mice at chow and high-fat diet, CRTC2 is phosphorylated at S136 by mTORC1 and mediates the effect of mTORC1 on SREBP-1 processing [128, 204]. Specifically, CRTC2 competes with SEC23 homolog A (Sec23A) to interact with Sec31A within the COPII complex, which is required for the transport of SREBP-1 from the ER to the Golgi [204]. Hepatic overexpression of the mTOR-defective mutant CRTC2(S136A) counteracts the unleashed SREBP-1 signaling in mouse liver on a high-fat diet and improves insulin sensitivity [204]. These findings indicate that CRTC2 plays a central role in controlling SREBP-1 signaling and lipid homeostasis in the liver. Less well understood is the extent to which CRTC2 regulates SREBP-1 and contributes to metabolic reprogramming in HCC. A recent report indicates that CRTC2 expression is induced in mouse liver by high-fat diet and activates the mTOR pathway by increasing miR-34a levels, reducing SIRT1-dependent deacetylation and downregulating TSC2 [220].

### TIP30 (HTATIP2, CC3)

The tumor suppressor TIP30 belongs to the short-chain dehydrogenases/reductases (SDR) family of more than 2000 NAD(H)/NADP(H)-dependent oxidoreductases and is involved in the regulation of cancer cell metabolism, survival, growth, and metastasis [221]. In the context of HCC, TIP30 deficiency has been shown to promote lipogenesis and cell proliferation in HCC cell lines and in a mouse xenograft model by enriching SREBP-1 in the nucleus through the Akt-mTOR pathway and inducing SREBP-1 and subsequent target gene expression [56]. Accordingly, TIP30-knockout mice are more prone to spontaneously develop HCC and other tumors as compared to wild-type mice [222]. The critical role of SREBP-1 regulation by TIP30 in tumorigenesis is further supported by the finding that TIP30 and SREBP-1 levels are inversely correlated in tumor samples from HCC patients [56]. Such an association is not observed for the surrounding non-tumorous area.

### Selected PI3K/Akt-regulatory pathways

Hepatic PI3K-Akt activation is not limited to insulin and growth factors [223]. Below, we exemplarily highlight three pathways that trigger the PI3K-Akt cascade and have been shown to regulate SREBP-1 in HCC.

#### CD147

The transmembrane glycoprotein CD147 is overexpressed in many human cancers, including HCC, and has attracted interest as a pharmacological target for the treatment of HCC [224]. Deletion of CD147 reduces the aggressiveness (i.e., tumor growth rate and metastasis) of human SMMC-7721 hepatocarcinoma cells in mouse xenografts, which is compensated by SREBP-1 overexpression [197]. Specifically, CD147 induces the expression of lipogenic enzymes (ACC1, FASN) by activating the AKT-mTOR-SREBP-1 pathway and represses enzymes involved in fatty acid β-oxidation (carnitine palmitoyltransferase 1 (CPT1A), peroxisome acyl-coenzyme oxidase 1 (ACOX1)) by activating p38 and downregulating PPARα [197]. In addition, N-acetylglucosaminyltransferase V (GnT-V)–mediated N-glycosylation of CD147 enhances its interaction with integrin β1 and stimulates HCC metastasis [225].

#### Thyroid hormones

The thyroid hormone 3,5,3′-triiodo-L-thyronine (T3) increases the expression of SREBP-1, apparently by binding to the integrin receptor αvβ3 and activating the PI3K-Akt-mTORC1 pathway in HepG2 cells, as suggested by inhibitor studies [86]. Protein kinase C-alpha (PKC-α) seems to act as a reverse regulator, dampening the T3-mediated upregulation of SREBP-1 [86]. 3,5-Diiodo-L-thyronine (T2), a precursor and by-product of thyroid hormone biosynthesis, instead activates p38 and extracellular signal-regulated kinase 1/2 (ERK1/2) and suppresses Akt signaling, which inhibits SREBP-1 maturation and promotes apoptosis in HepG2 cells [85].

#### Ephrin A3 (EFNA3)

Ephrins are membrane-bound ligands that bind to Eph receptors, which form the largest family of RTKs [226]. Signaling by Eph-ephrin can be forward, reverse, or bi-directional and does not necessarily require receptor-ligand interaction [226]. The Eph-ephrin system is involved in various developmental processes [226, 227]; is implicated in HCC metabolic plasticity, HCC malignancy, tumor progression, and aggressiveness (with mixed results regarding the directionality of the regulation) [45, 228, 229]; and inhibits PI3K-Akt-signaling [228]. EFNA3 is abundantly expressed in clinical HCC samples, and EFNA3 levels correlate with tumor aggressiveness [45]. EFNA3 expression is induced in HCC cell lines by HIF-1α under hypoxic conditions [45], which mimics the tumor microenvironment in hyperproliferative HCC *in vivo* [230, 231]. Through forward activation of EPH receptor A2 (EphA2), EFNA3 induces SREBP-1 maturation and expression of the SREBP-1 target gene ACLY, which mediates the self-renewal (cancer stemness–promoting) activity of the ephrin A3-EphA2-axis through metabolic rewiring [45]. Whether SREBP-1 activation is dependent on PI3K-Akt-signaling has not been explicitly investigated.

### Pharmacological interference

A large variety of small molecules interfere with SREBP-1 signaling by targeting SREBP-1 expression, maturation, degradation, post-translational modification, and upstream signaling cascades, such as PI3K, Akt, or mTOR. However, only a limited number of drugs, drug candidates, and natural products have been explicitly reported to inhibit SREBP-1 activity in HCC and HCC-related liver pathologies (Table 1). For example, bergapten (25–50 mg/kg/day, intraperitoneal administration (i.p.)), a furanocoumarin widely found in plants, suppresses the growth of N-nitrosodiethylamine (NDEA)-induced HCC in rats [58]. The anti-tumoral effects were correlated with increased LXRα/β expression (see the “LXR” section), suppressed fatty acid biosynthesis, impaired activation of the PI3K-Akt-SREBP-1 pathway, and reduced cholesterol uptake, likely due to a decrease in low-density lipoprotein receptor (LDLR) and an increase in cholesterol efflux through the upregulated ATP-binding cassette subfamily A member 1 (ABCA1) [58]. Another example is the anti-obesity drug orlistat, which increases the expression of PTEN, thereby inactivating Akt signaling and inhibiting SREBP-1 activation *in vitro* and *in vivo*. As a result, lipogenesis (as indicated by FASN expression) and cell proliferation are reduced in human hepatoma cell lines (20–50 μM orlistat), and steatosis and HCC progression are inhibited in rodent models (150–300 mg orlistat/kg/day; i.p.; 10–45 mg orlistat/kg/day, p.o.) [40, 124–126].

## AMPK

The serine-threonine kinase AMPK is an energy-sensor kinase that inhibits energy-consuming metabolic processes, including lipogenesis, and is downregulated in HCC patients [6, 107]. AMPK negatively regulates SREBP-1 transcription, proteolytic processing, nuclear translocation, and activation through multiple mechanisms, both directly through phosphorylation and indirectly by repressing pathways involved in SREBP-1 activation such as mTOR signaling [107]. Accordingly, hepatocyte-specific deletion of AMPK induced a mild steatotic phenotype in mice fed a high-fat/high-sucrose diet as compared to wild-type littermates [135]. In the context of liver physiology and HCC, AMPK activators such as metformin and 5-aminoimidazole-4-carboxamide ribonucleotide (AICAR) (Table 1) have been shown to negatively regulate hepatic transcription and processing of SREBP-1 and contribute to reduced HCC cell proliferation and liver tumorigenesis in mice [47, 79, 129–131].

Here, we briefly summarize the major mechanisms by which AMPK is considered to counteract SREBP-1 signaling in the liver. Firstly, AMPK phosphorylates S372 of SREBP-1c in mouse liver and inhibits SREBP-1c cleavage and nuclear translocation [134]. Insig1 is also directly phosphorylated by AMPK at T222, which ablates its interaction with gp78, stabilizes Insig1, and inhibits SREBP-1 cleavage and processing in mouse liver [135]. AMPK negatively regulates mTORC1 [107], which induces SREBP-1 signaling [201], and this mechanistic link has been proposed to contribute to the anti-tumoral activity of sorafenib in hepatocarcinoma cells [143]. By suppressing the biogenesis of circPRKAA1, a circRNA transcribed from the AMPK α1 subunit that interacts with Ku proteins (Ku80 and Ku70), AMPK decreases the stability of mSREBP-1 and reduces the development of tumors, including HCC [232]. Other proposed mechanisms by which AMPK interferes with SREBP-1 signaling include decreased cholesterol/LXR ligand biosynthesis, the inhibition of LXR-dependent SREBP-1c promoter activity, and diminished cleavage (maturation) of pSREBP-1c, which is likely to contribute to the suppressive effect on SREBP-1c transcription [233, 234]. A role for AMPK in suppressing SREBP-1 activity has also been described for the lipogenic activity of human HCC-associated protein TD26 (TD26). This oncoprotein is highly expressed in HCC tumor tissue (as compared to matched normal tissue), and its levels correlate with tumor size and poor prognosis in HCC patients [235]. TD26 interacts with SREBP-1 through its C-terminal domain and lowers AMPK-mediated suppression of SREBP-1 activity, thereby elevating lipogenesis and enhancing HCC cell proliferation and tumor growth [235]. In addition, AMPK enhances the activity of DNA methyltransferase 3A (DNMT3A), which maintains 5-methylcytosine (5mC) modifications at the thymine DNA glycosylase (TDG) promoter and diminishes TDG expression [79]. TDG is transactivated by c-Myc [79] and works in concert with members of the ten-eleven translocation (TET) family to oxidize 5-methylcytosine and cleave the product 5-carboxylcytosine (5caC) [236] from the SREBP-1 promoter (base-excision repair), among others, in hepatocarcinoma cells [79].

### Regulation of AMPK and link to ferroptosis

AMPK is regulated by a variety of signaling pathways [237]. Here, we briefly outline selected strategies used by HCC to control SREBP-1 signaling *via* AMPK and highlight the emerging link to ferroptosis, an alternative cell death program to apoptosis that is driven by excessive membrane peroxidation [113, 238].

The AMPK-SREBP-1c axis plays a central role in linking glucose and lipid metabolism in HCC [33]. For example, elevated exocellular lactate levels and lactate import activate the SREBP-1-SCD1 axis by repressing AMPK and promoting resistance to ferroptosis inducers [37]. Lactate accumulation is a hallmark of cancer cells, including HCC, and is attributed to the Warburg effect, which describes the preference of cancer cells for aerobic glycolysis over oxidative phosphorylation for energy production [239]. AMPK, SREBP-1c, and its target genes FASN and SCD1 determine susceptibility to membrane peroxidation and have been recognized as key players in ferroptosis [74, 240–246]. Accordingly, lactate depletion or inhibition of lactate import *via* the hydroxycarboxylic acid receptor 1 (HCAR1)/monocarboxylate transporter 1 (MCT1) impairs ATP generation and activates AMPK to repress SCD1, but also upregulates long-chain fatty acid CoA ligase 4 (ACSL4) [37], a lysophospholipid acyltransferase that preferentially channels PUFAs into membrane phospholipids [247]. The authors propose that both effects contribute to the induction of ferroptosis in HCC cells. Supplementation with SCD1 products, i.e., the MUFAs oleic acid or palmitoleic acid, actually compensates for the cytotoxicity induced by HCAR1/MCT1 suppression [37]. Note that lactate increases the levels of HCAR1, which is upregulated in tumors of HCC patients compared to adjacent tissues [37]. Together, lipid metabolic adaptations through the AMPK-SREBP-1c system essentially regulate ferroptosis sensitivity in HCC, as recently suggested for primary mouse embryonic fibroblasts and several cancer cell lines [37, 240, 248].

Another mechanism by which AMPK enhances ferroptosis sensitivity of HCC is related to branched-chain amino acid aminotransferase 2 (BCAT2), which was identified as a ferroptosis suppressor in a kinome CRISPR/Cas9-based screen in HepG2 cells [98]. BCAT2 catalyzes the reversible transamination of branched-chain amino acids and α-ketoglutarate, converting them to the corresponding α-keto acids and glutamate [98]. Note that the availability of glutamate is essential to keep ferroptosis at bay [238]. Accordingly, HCC cells are protected from lipid peroxidation when cellular glutamate levels and glutamate release are maintained [98]. Feedback loops seem to exist: the induction of ferroptosis in HepG2 cells has been proposed to trigger ferritinophagy and increase ROS levels, thereby activating AMPK, inhibiting SREBP-1, and downregulating BCAT2 [98].

There are also emerging links between AMPK and the ubiquitin-conjugating system, which interestingly has recently been implicated in the regulation of ferroptosis [249, 250]. In HCC cells, ubiquitin-conjugating enzyme E2 O (UBE2O) has been shown to confer malignant features, such as cell growth, migration, and invasion, by decreasing AMPKα2 stability and promoting the mTORC1 pathway [249]. UBE2O is overexpressed in HCC compared to adjacent normal tissues, and high UBE2O levels correlate with worse clinical outcomes in HCC patients [249]. Future studies are required to elucidate whether the UBE2O-dependent degradation of AMPKα2 along with mTORC1 activation effectively activates SREBP-1 in HCC and whether such regulation is functional in terms of ferroptosis sensitization and tumorigenesis.

Given the important role of inflammation and immune surveillance in HCC initiation and progression [230, 251–253], it is also noteworthy that HCC upregulates the expression of pro-inflammatory cytokines [254, 255]. TNFα and related cytokines promote tumor growth under certain conditions by creating a pro-inflammatory tumor microenvironment and/or initiating pro-survival signaling cascades [256, 257] and may also promote lipogenesis by interfering with AMPK activation (and other pathways) and relieving the repression of the mTORC1-SREBP-1c pathway, as shown for HCC cells [258, 259]. SREBP-1 itself has a dual role in inflammation in the tumor microenvironment, acting either to promote inflammation [38] or to resolve inflammation [39].

### Pharmacological interference

AMPK activators such as metformin [47, 79, 129–141, 260–268] and AICAR [98, 132, 133, 135, 139, 142, 262, 269–271] have been extensively studied in liver pathologies, including HCC, and have been shown to suppress lipogenesis and/or tumorigenesis in pre-clinical and clinical studies (Table 1). Note that metformin activates AMPK in the liver through direct interaction with PEN2, a subunit of γ-secretase, resulting in inhibition of v-ATPase and activation of AMPK [264], but also has AMPK-independent activities [266, 272, 273]. Although a negative regulation of SREBPs is widely expected, only a fraction of these studies provided experimental evidence to support that AMPK inhibitors interfere with SREBP-1 signaling in HCC [47, 129, 131, 135, 136].

The multikinase inhibitor sorafenib is in clinical use for the treatment of HCC [274, 275]. Among many other activities, sorafenib has been shown to activate the cellular energy sensor AMPK by decreasing ATP production [143]. Subsequent phosphorylation of TSC2 at S1387 [276] and the mTORC1 subunit Raptor at S722 and S792 inhibits mTORC1-mediated SREBP-1 activation [277], suppresses the expression of the SREBP-1 target gene SCD1, and reduces the availability of MUFAs in Huh7 cells [143, 278]. Interestingly, sorafenib-resistant HCC cell lines highly express programmed death ligand 1 (PD-L1), which activates the PI3K-Akt-SREBP-1 axis in HCC cell lines and installs features that are characteristic of EMT [72]. Accordingly, high levels of SREBP-1 in tumor tissue of HCC patients receiving sorafenib correlate with poor prognosis [66], which suggests that SREBP-1 contributes to the resistance of HCC cells to sorafenib. In support of this hypothesis, the knockdown of SREBP-1 synergizes with the tumor-suppressive activity of sorafenib against HCC [66].

Diverse natural products have been reported to activate AMPK and suppress SREBP-1 in HCC (Table 1). For example, the polyphenol resveratrol (30 mg/kg/day, p.o.) protects against hepatitis B virus (HBV)–induced fatty liver and HCC (incidence reduced from 80 to 15%) in HBV X protein (HBx) transgenic mice. Resveratrol represses major enzymes in *de novo* fatty acid biosynthesis, i.e., ACC and FASN, seemingly by inhibiting LXRα and downregulating SREBP-1 and PPARγ and activating AMPK and SIRT1 [147]. In addition, resveratrol promotes transient liver regeneration and stimulates the cellular antioxidant response, as indicated by elevated intracellular glutathione levels [147]. Along these lines, piceatannol (3,3′,4,5′-tetrahydroxy-trans-stilbene) (at 100 μM), a stilbenoid metabolite of resveratrol, suppresses the mRNA expression of SREBP-1c and PPARγ, while inducing PPARα in HepG2 cells, which is associated with an apparent shift from anabolic to catabolic fatty acid metabolism [191]. Based on previous studies on resveratrol and piceatannol suggesting activation of AMPK in liver disease [279], the authors propose that the two polyphenols employ similar AMPK-dependent mechanisms to attenuate lipid accumulation [191]. Other examples include isoquercitrin (at 10–200 μM) [148–150], the tetracyclic triterpenoid limonin (50–200 μM; 50 mg/kg/day, p.o.) [155], and curcumin (1–10 μM; 80–100 mg/kg/day, p.o.) [151, 152], which promote the phosphorylation of AMPKα, decrease SREBP-1 expression, downregulate lipogenic proteins or interfere with their activation, and reduce total lipid content in HCC cell lines [151]. Inhibitor studies for isoquercitrin confirmed a functional link between AMPK and ACC and adiponectin receptor 1 (AdipoR1) expression [148]. Furthermore, the pentacyclic triterpenoids tormentic acid (0.06–0.12 g/kg/day, p.o.) and betulinic acid (5–10 mg/kg, p.o.) and the pentacyclic triterpene saponin pedunculoside (5–30 mg/kg/day, p.o.) inhibit high-fat diet–induced hepatic lipid accumulation [127, 160, 169] and hyperlipidemia [127, 169] in rodents along with increasing AMPK phosphorylation and reducing SREBP-1 expression. Similar mechanisms seem also to apply to distinct plant extracts with hepatoprotective properties. For example, mulberry anthocyanin extract (at 300–500 μg/ml) and fermented Rhus verniciflua Stokes extract (at 200–400 μg/ml) inhibit triglyceride and/or cholesterol accumulation in HepG2 cells that were overloaded with oleic acid [280, 281], which has been ascribed to enhanced AMPK phosphorylation and decreased SREBP-1 expression.

## PKA

The serine-threonine kinase cAMP/cAMP-dependent protein kinase A (PKA) is regulated by various stimuli that activate G-protein-coupled receptors (GCPRs) or interfere with the adenylate cyclase/phosphodiesterase system to modulate the concentrations of the second messenger cAMP, which activates PKA [83]. Among many other functions, PKA is a critical signaling kinase in the control of glucose and lipid metabolism, converging signal transduction by GCPR ligands, including insulin and glucagon [83]. PKA plays a central role in suppressing lipogenesis, among others, through pathways that negatively regulate SREBP-1 [83]. By directly phosphorylating human SREBP-1a at S338 and SREBP-1c at S314, PKA suppresses transcriptional activity [282]. In addition, PKA represses the expression of SREBP-1c by activating the FXR or SIRT1 [83] and by phosphorylating AMPKα1 at S173, which inactivates the kinase by impeding T172 phosphorylation [283]. Of relevance to liver metabolism and HCC, glucagon triggers cAMP generation and has opposite effects compared to insulin, among others, by preventing insulin-induced SREPB-1c expression and maturation [80]. cAMP-activated PKA phosphorylates SREBP-1a, thereby attenuating their DNA occupancy in cell-free assays and in HepG2 cells [284]. On the other hand, PKA phosphorylates PPARα, which induces SREBP-1c transcription and likely explains why hepatic lipid accumulation is increased in TSH-deficient mice on chow and high-fat diet [84]. Conclusively, cAMP-PKA exerts both lipogenesis-promoting and lipogenesis-inhibiting activities by regulating SREBP-1 in HCC depending on the stimulus and context.

## LXR

LXRs (LXRα and LXRβ) are nuclear hormone receptors that form heterodimers with retinoid X receptor (RXR) and are activated by oxysterols and several exogenous ligands [285]. By transactivating metabolic drivers, such as SREBP-1c and ABCA1, LXRs regulate cholesterol homeostasis and lipogenesis [285, 286]. While LXRβ is ubiquitously expressed, LXRα is most abundant in metabolic tissues, including liver [285]. Various metabolic signaling pathways that regulate SREBP-1 signaling cross-talk with LXRs. For example, PPARα and PPARγ (see the “PPARs” section) directly bind to and transactivate the LXRα promoter [287, 288], and PI3K-Akt-mTORC1 activation by insulin or growth factors (see the “PIAK-Akt-mTOR axis” section) stimulates SREBP-1c transcription through diverse mechanisms, some of which involve LXR [77, 289]. Endogenous ligands that antagonize LXR activity include PUFAs, such as arachidonic acid and docosahexaenoic acid [290] (see the “PUFAs” section). Note that LXRs are upregulated in various cancers and that LXR agonists have been shown to counteract tumorigenesis and metastasis in experimental cancer models [285].

In the context of HCC, LXRα/β and its heterodimeric partner RXR target two LXR binding elements (LXREs) at the SREBP-1c promoter and conjointly activate transcription, as shown by LXR and RXR transfection and pharmacological approaches using the natural LXR ligand 22(R)-hydroxycholesterol and the RXR ligand 9-cis-retinoic acid [10, 291]. Accordingly, both total and nuclear SREBP-1c levels increase along with FASN expression. The observation that LXR activation induces SREBP-1 mRNA expression and increases mSREBP-1 levels was independently confirmed in HepG2 cells using the selective LXR ligand T0901317 [292].

The activity of LXR is inhibited by a short heterodimer partner (SHP) in rat hepatoma McA-RH7777 cells, likely through suppressive heterodimerization that reduces SREBP-1 expression [97]. SHP is an atypical member of the nuclear hormone receptor family, which possesses a putative ligand-binding domain but lacks a conventional DNA-binding domain [293]. Activation of FXR induces the expression of SHP, which explains the triglyceride-reducing activity of natural FXR ligands such as bile acids [97]. In fact, a bile acid (cholic acid)–rich diet decreases SREBP-1 promoter activity, diminishes hepatic mRNA expression of SREBP-1c and other lipogenic genes, and lowers plasma and hepatic triglyceride levels in mouse models of hypertriglyceridemia [97].

## PPARs

The three PPAR isotypes PPARα, PPARβ/δ, and PPARγ form heterodimeric complexes with RXR and act as nuclear receptors for endogenous ligands, such as fatty acids and their derivatives [294, 295]. PPARα is highly expressed in the liver and is targeted by fibrates, lipid-lowering drugs used to treat dyslipidemia, whereas PPARγ is the target of thiazolidinediones, anti-diabetic drugs that sensitize extrahepatic tissues to insulin [295]. PPARs have both positive and negative effects on the SREBP-1 pathway [296, 297]. For example, PPARα induces SREBP-1c transcription by binding directly to the DR1 element at the SREBP-1c promoter in human liver [288], but also upregulates Insig2α expression during fasting, thereby suppressing SREBP-1c processing [297]. PPARδ promotes Insig1 expression by binding upstream of the transcription initiation site and suppresses SREBP-1 proteolytic processing in the liver of obese diabetic mice [296]. With respect to HCC, both PPARα and PPARγ activation have been shown to reduce nuclear SREBP-1 availability, inhibit SREBP-1 target gene expression, and decrease triglyceride production in rat hepatoma Fao cells [298]. In the previous section “PKA,” we have already mentioned the regulation of SERBP-1 by PPARs as part of or in parallel with other SREBP-1-regulatory pathways (cAMP-PKA, LXRα).

## PGC-1

The induction of SREBP-1c (and to a lesser extent SREBP-1a) transcription by LXR is amplified (in HepG2 cells) by peroxisome proliferator-activated receptor γ co-activator 1α (PGC-1α), which acts as a co-activator of LXR [299]. PGC-1β also co-activates SREBPs, contributing significantly to the SREBP-dependent lipogenic gene expression in mouse H2.35 hepatoma cells and rat liver [300]. PGC-1β physically interacts with SREBP-1c through a specific domain (amino acids 350–530) that is absent in PGC-1α. Despite the induction of lipogenic SREBP-1c target genes in PGC-1β-transgenic rats, hepatic triglyceride and cholesterol levels were markedly decreased, seemingly due to the co-activation of LXR by PGC-1β and stimulation of lipoprotein secretion [300].

Through a PGC-1-dependent mechanism, pharmacological inhibition of p38 increases insulin-induced expression of SREBP-1 target genes in primary hepatocytes [82]. p38 inhibition also induces triglyceride accumulation in mouse liver on high-fat diet and reverses the decrease in triglyceride levels along with the expression of SREBP-1 and lipogenic target enzymes, as shown in glucagon-treated primary hepatocytes (see the “PKA” section) and in fasting mouse liver [82]. Mechanistically, p38 attenuates the insulin-induced increase as well as the fasting- and glucagon-induced decrease in PGC-1β transcription [82], which inhibits the co-activation of SREBP-1 transcription [300]. Together, p38 negatively regulates SREBP-1-dependent hepatic lipogenesis through the PGC-1β-SREBP-1c pathway. The regulatory function of p38 on SREBP-1 promoter activity was confirmed in mouse Hepa1c1c7 hepatoma cells [82].

## STAT

Janus kinase (JAK)/STAT inhibitors have been extensively studied in the context of liver pathology and are currently under clinical investigation for the treatment of HCC [301]. JAKs are associated with various cytokine and growth factor receptors and, when activated, phosphorylate intracellular receptor domains to recruit STAT proteins (STAT1-6). STATs are phosphorylated by JAKs, dimerize, and act as transcription factors to regulate the expression of specific genes involved in immunoregulation, inflammation, cell growth, survival, differentiation, and adipogenesis [301]. They are expressed in a cell type–dependent manner and induce both overlapping and unique responses. Cross-talk exists between JAK-STAT signaling and various pathways at the intersection of metabolic control and cancer, including the PI3K-Akt-mTOR axis, transforming growth factor β (TGFβ) signaling, and Notch signaling [301].

The functional link between STAT signaling and SREBP-1 is still emerging. Of relevance to HCC, mTORC1 interacts with and phosphorylates STAT5, which then binds to the SREBP-1 promoter and stimulates SREBP-1 transcription [8]. Accordingly, liver-specific activation of mTORC1 increases lipogenic gene expression and spontaneous tumorigenesis in mouse liver [8], and high levels of SREBP-1 and phospho-STAT5 are associated with poor survival in HCC patients [8]. Furthermore, liver-specific STAT5 knockout in mice induces steatosis by upregulating SREBP-1 and PPARγ signaling and simultaneously activating the c-Jun N-terminal kinase 1 (JNK1)-STAT3 pathway [87]. When liver-specific STAT5 deficiency is combined with glucocorticoid receptor deletion, fatty liver develops into HCC [87]. This malignant transformation is ascribed to lipid mobilization from adipose tissue and aggravated hepatic lipid accumulation along with insulin resistance and increased expression of the pro-inflammatory cytokine TNF-α [87]. Another study found that TNF-α and insulin upregulate proprotein convertase subtilisin/kexin type 9 (PCSK9) in HepG2 cells dependent on suppressor of cytokine signaling 3 (SOCS3)-JAK-STAT3 [92]. Overexpression of SOCS3 induces Akt phosphorylation and increases the expression of SREBP-1c, lipogenic genes, and ApoB, without affecting cholesterol biosynthesis [92]. Whether PCSK9 contributes to the regulation of SREBP-1 in addition to its role in insulin resistance has not been investigated. ROS-scavenging and inhibitor studies in high glucose–challenged HepG2 cells suggest that PCSK9 expression is induced by ROS-dependent enrichment of nuclear SREBP-1 (but not SREBP-2) [302]. Indeed, oxidative stress activates SREBP-1c and induces lipid accumulation in HepG2 cells [303], and an SRE binding site exists on the PCSK9 promoter [81]. The authors further propose that PCSK9 elevates extracellular levels of LDL cholesterol (LDLc), which protects against sorafenib-induced HCC cell death [302].

Notably, the hepatoprotective flavonolignan silibinin from *Silybum marianum* inhibits STAT3 activation and suppresses SREBP-1-mediated lipid accumulation in endometrial carcinoma cells and tumors [304, 305]. Although a functional link between STAT3 and SREBP-1 signaling has not been confirmed for the anti-tumoral effect of silibinin, we consider such a mechanism likely to contribute to the proposed anti-lipogenic and HCC-preventive activities of the natural extracts.

## MYC

The proto-oncogenic transcription factor Myc is induced in response to mitogenic stimuli; regulates genes involved in cell metabolism, growth, and proliferation [306, 307], among them SREBF1; and is frequently overexpressed in cancer, including HCC [306, 307]. Direct binding of Myc to two recognition sites on the SREBP-1 promoter has been confirmed by chromatin immunoprecipitation [308]. Furthermore, N-Myc, a member of the Myc protooncogene family, induces SREBP-1 expression (along with the reprogramming of other metabolic pathways) dependent on the Myc superfamily transcription factor MondoA, which acts as a nutrient sensor [309]. Myc and SREBP-1 cooperate in the expression of lipogenic target genes and drive lipogenesis and tumorigenesis in several cancer models [310]. While Myc and SREBP-1 have been independently described to promote HCC in different systems [7, 10, 11, 306, 307], studies demonstrating that Myc induces SREBP-1 expression in HCC are rare [310]. Insulin-induced activation of c-Myc has been reported to transactivate TDG [79], which mediates hypomethylation of the SREBP-1 promoter, promotes lipogenesis, and induces the proliferation of hepatocarcinoma and other cancer cell lines [79]. In further support of Myc regulating SREBP-1 in HCC, the acyl-CoA synthetase isoenzyme ACSL4 was recently shown to promote SREBP-1 expression by stabilizing Myc in hepatoma cells [308, 311], as described in the “PUFAs” section.

## Kinases that directly phosphorylate SREBP-1

Diverse kinases phosphorylate and thereby regulate the activity, subcellular localization, maturation, and stability of SREBP-1 [134, 214, 284]. In the context of HCC, SREBP-1 has been reported to be directly phosphorylated by GSK3, AMPK, and PKA [134, 214, 284], as discussed in the “Protein kinases” section, “AMPK” section, and “PKA” section. mSREBP-1 is also directly phosphorylated at S439 and thereby stabilized by the cyclin-dependent kinase 1 (Cdk1)/cyclin B complex [312], which coordinates G2/M progression [313]. The authors conclude that this phosphorylation might help preserve basal lipogenesis during and beyond mitosis to support cell growth and proliferation [312]. Another kinase linking glucose metabolism to SREBP-1 activation is the pyruvate kinase isoenzyme M2 (PKM2), which catalyzes the final step of glycolysis and is overexpressed in different cancer types, including HCC, but also has complex functions related to redox homeostasis and inflammation [314–319]. PKM2 phosphorylates mSREBP-1a at T59, thereby promoting interaction with PKM2, stabilizing mSREBP-1a, stimulating HCC cell proliferation, and correlating with poor prognosis of HCC patients [181]. Notably, Zhao et al. were able to block the interaction of PKM2 and mSREBP-1a and thereby attenuate lipogenic gene expression using a competitive peptide inhibitor corresponding to the amino acid sequence of SREBP-1a from 43-56 [181].

## Histone acetyltransferases and sirtuins

The expression of SREBP-1 is controlled by post-translational acetylation, with the histone acetyltransferase CREB-binding protein/E1A binding protein p300 (CBP/p300) and sirtuins (SIRT), a family of NAD^+^-dependent deacetylases, dominating the regulation of SREBP-1 in HCC [182, 320, 321]. Histone acetyltransferases CBP/p300 activate the transcriptional activity of SREBP-1c by acetylating the transcription factor at lysine-289 (K289) and K309, which promotes the accessibility to target gene promoters and enhances SREBP-1c stability by suppressing ubiquitination-dependent degradation [320].

The seven members of the SIRT family are class III histone deacetylases that differ in subcellular localization and functions related to cell metabolism, cancer, and many other physiological and pathophysiological processes [322]. SIRT1 directly interacts with SREBP-1c, decreases the acetylation at K289 and K309, and inhibits the lipogenic activity of SREBP-1c in mouse liver by reducing its stability and occupancy at target gene promoters [182]. The interaction of SIRT1 with SREBP-1c is enhanced during fasting and reduced during feeding (insulin/glucose), reflecting changes in SREBP-1c acetylation [182]. SIRT1 is regulated by manifold pathways and cellular processes, such as the DNA damage and oxidative stress response [322, 323]. In HCC, activation of SIRT1 is vital for cancer progression and has been closely linked to mitochondrial fission [321, 324], a tightly regulated process used to meet the metabolic demands of the cell [325]. Increased mitochondrial fission decreases cellular NAD^+^ levels and SIRT1 activity in HCC cells, which increases the acetylation of SREBP-1 and PGC-1α [321]. As a result, lipogenic SREBP-1c target enzymes (FASN, ACC1, and ELOVL6) are upregulated, while the expression of enzymes involved in fatty acid oxidation (CPT1A and ACOX1, which are under the control of PGC-1α/PPARα) is inhibited. This reprogramming of lipid metabolism is associated with lipid accumulation (fatty acids, triglycerides, phospholipids) and increased metastasis [321].

SIRT6 inhibits the transcription of SREBP-1 and lipogenic target genes and seems to be regulated in HCC by the nuclear envelope protein sperm-associated antigen 4 (SPAG4) [125]. Since SPAG4 binds to lamin A/C with higher affinity than SIRT6, the authors speculate that the competitive binding of SPAG4 to lamin A/C releases SIRT6 and increases the expression, nuclear translocation, and transcriptional activity of SREBP-1. SPAG4 is highly expressed in HCC, and its levels correlate with poorer survival in HCC patients [125]. In support of a functional role of SREBP-1 in SPAG4-driven HCC progression, an interference with SREBP-1 signaling by orlistat attenuates tumor growth in mouse xenografts and synergizes with sorafenib [125]. In addition, SIRT6 activation by fluvastatin (an HMG-CoA reductase inhibitor) increases SIRT6 expression and stimulates inhibitory SREBP-1 phosphorylation *via* AMPK in HepG2 cells, thereby decreasing lipid biosynthesis, as expected [170].

SIRT7 binds to SREBP-1 and maintains the expression of isocitrate dehydrogenase 1 (IDH1) in a SREBP-1-dependent but SIRT7 deacetylase activity-independent manner in HCC cell lines [34]. IDH1 utilizes NADP^+^ and converts isocitrate to α-ketoglutarate and NADPH by oxidative decarboxylation but also catalyzes the reverse reaction and contributes to lipid biosynthesis from glutamine in several cancers [326–330]. Silencing of SIRT7 accordingly reduces lipogenesis and gluconeogenesis, as expected from interference with the (seemingly stimulatory) interaction of SIRT7 with SREBP-1, which sustains cellular IDH1 levels [34].

## Further post-translational modifications of SREBP-1

In addition to phosphorylation (“Kinases that directly phosphorylate SREBP-1”), acetylation (“Histone acetyltransferases and sirtuins”), and ubiquitination (“PI3K-Akt-mTOR axis”), SREBP-1 has been reported to undergo methylation and conjugation with arginine methyltransferase 5 (PRMT5) and neddylation by NEDD8-conjugating E2 ligase (UBC12) in HCC cells [65, 331]. PRMT5 symmetrically dimethylates SREBP-1a at arginine-321 (R321) in HepG2 cells, which prevents destabilizing phosphorylation at S430 by GSK3β and thereby attenuates the recruitment of the ubiquitin ligase FBW7 [331]. Thus, SREBP-1a methylation promotes lipogenesis and HCC cell growth/proliferation *in vitro* and *in vivo* and correlates with tumor progression in HCC patients [331].

NEDD8 (neural precursor cell expressed developmentally downregulated protein 8) is a ubiquitin-like protein that is covalently attached to substrate proteins by the ubiquitylation machinery and regulates various signaling pathways besides cullins and other non-cullin ubiquitin E3 ligases [332]. Neddylation of SREBP-1 is mediated by UBC12 in HepG2 and grafted SK-Hep1 cells (a human endothelial-like cell line derived from HCC) [65]. Conjugation with NEDD8 stabilizes SREBP-1 (which is less ubiquitylated) and correlates with poor metastatic tumor prognosis in HCC. Selective inhibition of neddylation by MLN4924 (an inhibitor of Nae1) (30 mg/kg, twice daily, subcutaneous administration (s.c.)) in SK-Hep1 mouse xenografts indeed reduces SREBP-1 levels, lipogenesis, and tumor growth while inducing cell death [65]. Note that proteomic and electron microscopic data suggest that SK-Hep1 cells are of endothelial and not hepatocytic origin, although they have been widely used as a cell model for HCC [333].

## Sterols and oxysterols

As described in the “SREBP-1” section, SREBP-1/2 maturation responds to cellular metabolic demands and is induced by insufficient availability of sterols, such as cholesterol or 25-hydroxycholesterol [99, 334–336]. In the context of HCC, dietary cholesterol suppresses tumorigenesis by reducing SCAP-dependent *de novo* lipogenesis, whereas cholesterol deprivation has the opposite effect, as shown for DEN-induced HCC in mice [55]. Accordingly, overexpression of the cholesterol-biosynthetic enzyme NAD(P)-dependent steroid dehydrogenase-like (NSDHL) decreases the levels of active nuclear mSREBP-1 in HCC cell lines, whereas silencing of the enzyme increases mSREBP-1 activation [73]. The authors suggest that the SREBP-1-dependent expression of the immunosuppressive cytokine TGFβ contributes to resistance to immune checkpoint inhibitors. Indeed, immune checkpoint inhibitors activate tumor antigen–specific T cells to produce IFN-γ [337], and IFN-γ decreases NSDHL expression and stimulates SREBP-1 maturation in HCC with subsequent release of TGFβ [73]. Activation of SREBP-1/2 is also closely related to the activity of other steroid biosynthetic enzymes, including cholesterol 7α-hydroxylase (CYP7A1) [97, 335] and oxysterol 7α-hydroxylase (CYP7B1) [338], which catalyze the first step in bile acid biosynthesis by converting cholesterol to 7α-hydroxycholesterol [339] and metabolize 25- and 27-hydroxycholesterol, respectively [339]. For example, overexpression of CYP7A1 in rat hepatoma McArdle cells lowers cellular cholesterol levels, enhances SREBP-1 activation, and induces the expression of enzymes involved in fatty acid and sterol biosynthesis, along with increased cellular lipid synthesis and ApoB100 secretion [335]. Treatment with 25-hydroxycholesterol (which inhibits proteolytic processing of SREBP-1) reverses the CYP7A1-mediated increase in mSREBP-1 levels and increases the secretion of ApoB100 [22, 335]. CYP7B1 and SREBP-1 activation are mutually regulated, with SREBP-1 repressing CYP7B1 transcription in rat McA-RH7777 hepatoma cells, likely by interacting with the basal transcriptional activator Sp1 at GC-rich sequences within the proximal promoter of CYP7B1 [338].

## PUFAs

PUFAs are considered to compete with agonistic LXR ligands for binding to the LXR ligand-binding domain, thereby preventing the LXR/RXR heterodimer from interacting with the SREBP-1c promoter and inhibiting SREBP-1 expression [100]. In addition, independent of LXR, PUFAs suppress the proteolytic processing of SREBP-1 in mouse liver, thus disrupting the autoregulatory feedback loop by which SREBP-1 induces its own expression [99]. PUFAs also accelerate SREBP-1 mRNA degradation and, as shown for docosahexaenoic acid, reduce mSREBP-1 stability and nuclear availability through ERK- and 26S proteasome–dependent pathways, among others, in hepatocytes [101, 340].

Negative regulation of SREBP-1 by PUFAs is critical for hepatic lipid homeostasis, as exemplified by the deletion of ELOVL5 in mice [25]. ELOVL5 is involved in the conversion of essential fatty acid precursors (i.e., linoleic acid, linolenic acid) to arachidonic acid, docosapentaenoic acid, and docosahexaenoic acid, and its knockout reduces the hepatic availability of these PUFAs [341]. As a result, SREBP-1c is activated, triggering the expression of lipogenic target genes and inducing hepatic steatosis [25]. The authors propose a feedback loop whereby PUFA biosynthesis, enhanced by SREBP-1, interferes with the activation of this transcription factor [25]. A population-based investigation supports that ω3 PUFA intake through fish consumption or ω3-fatty acid supplementation reduces the risk of HCC [342]. Dietary ω6 PUFAs, by contrast, show a dose-dependent, positive association with HCC risk [343]. However, it remains unclear whether PUFAs suppress SREBP-1 activation in the human liver and whether this mechanism contributes to the proposed anti-HCC activity of ω3-fatty acids.

In HepG2 cells treated with the LXR agonist T0901317, oxidized PUFAs (i.e., the oxidized ω3-fatty acid eicosapentaenoic acid) possess superior anti-lipogenic activity over non-oxidized fatty acids, and this metabolic shift correlates with decreased SREBP-1c and PGC-1β expression [344]. Further studies are needed to clarify whether this gain in inhibitory activity compensates for the lower cellular concentrations of oxidized PUFAs relative to parental levels and whether PUFA oxidation has pathophysiological relevance in HCC. Importantly, oxygenated PUFAs are precursors of structurally diverse lipid mediators that combine potent pro-inflammatory, anti-inflammatory, pro-resolving, and immunomodulatory properties with tumor- and metastasis-regulatory functions [30, 31, 345]. Peroxidation of phospholipid-bound PUFAs, on the other hand, is a hallmark of ferroptosis, the induction of which by small molecules is currently being explored as a potential strategy against (therapy-resistant) HCC [346].

Another study in human Huh-7 and SMMC-7721 hepatoma cells silenced ACSL4 [308], an isoenzyme that couples fatty acids to coenzyme A and preferentially accepts PUFAs, which can then be incorporated into triglycerides, phospholipids, or CE esters [247]. ACSL4 is an oncogenic marker of the α-fetoprotein-high subtype of HCC and promotes HCC tumor formation and metastasis in Huh-7-grafted mice [308]. The tumor-promoting activity is partially dependent on SREBP-1, as demonstrated by SREBP-1 overexpression in ACSL4-silenced tumors. ACSL4 enhances SREBP-1 expression by stabilizing c-Myc [311], which binds directly to the SREBP-1 promoter region and activates transcription [308]. In consequence, the expression of the SREBP-1 target genes is induced and triglycerides and cholesterol accumulate. Mechanistically, ACSL4 attenuates proteasomal degradation of c-Myc in an ERK- and FBW7-dependent manner [311]. How ACSL4 modulates the ERK-FBW7-c-Myc axis is not readily understood, though the mechanism likely involves changes in the availability of PUFAs or PUFA-derived metabolites.

Complementary mechanistic insights come from the genetic manipulation of lysophosphatidylcholine acyltransferase 3 (LPCAT3) in primary mouse hepatocytes and liver [347]. The LXR target gene LPCAT3 uses PUFA-CoA (derived from ACSL4) as substrate and incorporates the acyl-chain into phosphatidylcholine (PC), phosphatidylethanolamine (PE), and phosphatidylserine (PS) [348, 349]. Accordingly, LPCAT3 deletion decreases the proportion of PUFA-containing PC in the ER, reduces SREBP-1 processing to the mature nuclear form, and thereby suppresses lipogenic responses [349]. (i) Feeding, (ii) delivery of exogenous PUFA-containing PC to the ER, and (iii) LXR activation have opposite effects [349]. Dynamic changes in the PUFA composition of the ER membrane influence either the SCAP/SREBP-1 interaction or the transport of SREBP-1 to the Golgi [348]. The detailed mechanisms remain elusive and might involve phospholipid classes other than PC. In support of this hypothesis, PE with saturated fatty acids impairs the processing of the Drosophila SREBP ortholog in S2 cells [350], and inhibition of *de novo* phospholipid biosynthesis leads to a mislocalization of S1P and S1P to the ER (instead of the Golgi), allowing efficient processing of SREBP-1 [351]. Interestingly, supplementation of PUFA-containing PC and depletion of LPCAT3 have also been reported to decrease Akt phosphorylation by reducing the kinase’s affinity to phosphoinositides at membranes [352, 353]. Whether the associated decrease in long-term Akt signaling affects SREBP-1 expression has not been addressed.

## Other SREBP-1 regulatory factors and mechanisms

In the following, we summarize diverse mechanisms that have been shown to regulate SREBP-1 in HCC and discuss their (pre-)clinical implications.

The multidomain adaptor protein β2-spectrin (SPTBN1), which is involved in TGFβ-SMAD3 (mothers against decapentaplegic homolog 3) signaling, among others, increases SREBP-1 activity, lipogenesis, and HCC progression in mice fed a high-fat diet or a Western diet [95]. The authors propose that caspase 3 cleaves SPTBN1 and SREBP-1 and that the N-terminal product of SPTBN1 (N-SPTBN1) interacts with cleaved SREBP-1 to stabilize the nuclear form of SREBP-1, thereby inducing the expression of target genes. Accordingly, liver-specific deletion of SPTBN1 (which is overexpressed in NASH along with caspase 3) protects mice against hepatic steatosis, fibrosis, inflammation, tissue damage, and HCC [95].

MicroRNA-27a (miR-27a) modulates cancer cell proliferation, apoptosis, migration, and invasion as well as angiogenesis and therapy resistance and has both oncogenic and tumor suppressor functions [354–358]. miR-27a is also preferentially expressed in HCV-infected liver and has in this context been found to repress SREBP-1 expression along with other major lipogenic regulators and enzymes, including RXRα, PPARα, PPARγ, and FASN in human HUH-7.5 hepatocellular carcinoma cells [359]. Repression of miR-27a increases cellular lipid accumulation and HCC infectivity, whereas overexpression has the opposite effect.

The related RNA binding proteins Lin28A and Lin28B (Lin28A/B) are upregulated in HCC and other cancers [360]. They initiate the post-transcriptional repression of the let-7 microRNA family, but also bind various mRNAs with roles in cell metabolism, cell cycle progression, and survival [361–365]. Among these mRNAs are those of SREBP-1 and SCAP, which are positively regulated by Lin28A/B in HCC cell lines [43]. By interacting with SREBP-1 and SCAP mRNA, the proto-oncogenes Lin28A/B enhance the translation and processing of SREBP-1 and stimulate tumor growth in mouse xenografts of human PLC hepatocellular carcinoma cells [43]. Mechanistically, the authors (i) demonstrate that SREBP-1, the SREBP-1 target gene SCD1, and ER stress (or the associated UPR) are involved in HCC progression; (ii) point to the imbalance of fatty acid unsaturation upon Lin28A/B silencing; and (iii) propose that Lin28A/B protects HCC cells from ER lipotoxicity [43]. Whether the recently discovered SCD1-derived lipokine PI(18:1/18:1) is enriched upon SCD1 upregulation and contributes to the tumor-protective activity of Lin28A/B has not been addressed [366].

HDGF has a highly conserved N-terminal PWWP domain and exerts oncogenic activity (related to transformation, survival, metastasis, and angiogenesis) through incompletely understood mechanisms [367–371]. Nuclear-localized HDGF was recently shown to bind to and act as a co-activator of SREBP-1 and to enhance lipogenic gene expression in HepG2 cells by attenuating the recruitment of the transcription repressor C-terminal binding protein 1 (CTBP1) [89]. In support of a functional role of (nuclear) HDGF in regulating SREBP-1 levels, the combined expression of the two factors indicates a poor prognosis in HCC [89]. Sequence variability exists in PWWP, and the A-type variant (in contrast to wildtype HDGF) recruits CTBP1, suppresses lipid biosynthesis, and decreases proliferation of HepG2 cells, both *in vitro* and in murine xenografts [89].

The transcription factor p53, a major tumor suppressor, and ferredoxin reductase (FDXR), which is involved in steroid biosynthesis, negatively regulate the maturation of SREBP-1/2 and thus keep cellular levels of triglycerides and cholesterol in check, as shown for mouse embryonic fibroblasts (MEFs), HepG2 cells, and/or mouse liver [372]. The authors further suggest, based on correlative data, that the availability of the cholesterol efflux pump ABCA1, which is induced by either p53 [373] or FDXR [372], determines SREBP-1/2 activation [372]. Deletion of p53 or FDXR (as well as the double KO) induces hepatic steatosis, inflammation, and spontaneous tumorigenesis in mice, which is, however, not limited to HCC [372]. On the other hand, p53 binds to the SREBP-1 promoter and induces SREBP-1 transcriptional activity in HepG2 cells [46]. NAD(P)H quinone oxidoreductase-1 (NQO1), which is highly expressed in HCC and associated with poor outcome, induces SREBP-1 transcription in HepG2 cells through this mechanism, specifically by preventing ubiquitination and proteasomal degradation of p53 [46]. Notably, the NQO1-p53-SREBP-1-axis stabilizes the EMT transcription factor Snail, thereby inducing lipid anabolism and EMT of HCC cells, which promotes the progression and metastasis of HCC [46].

## Inhibitors of SREBP-1 signaling with diverse mechanisms

A large number of small molecules and some oligonucleotide-based approaches have been reported to interfere with SREBP-1 signaling in HCC (respectively HCC-promoting liver pathologies) by different, only partially understood mechanisms, as summarized in Table 1 and discussed below for selected compounds.

1-(4-Bromophenyl)-3-(pyridin-3-yl)urea (SI-1), an inhibitor of SREBP-1 activation, decreases the mRNA expression of SREBP-1 target genes in HCC cells more potently than betulin or fatostatin (FASN: IC_50_ = 0.3, 1.6, and 1.0 μM, respectively), lowers aerobic glycolysis, and potentiates the anti-tumoral efficacy of radiofrequency ablation towards xenograft HCC (at 0.5–5 mg/kg; peroral administration, p.o.) [44].

SREBP decoy oligodeoxynucleotides are short, double-stranded DNA sequences that mimic SREs and compete with them for binding to SREBP without generating a functional response, thereby blocking the expression of SREBP target genes [374]. They have been shown to inhibit the expression of SREBP-1c, ACC1, FASN, SCD1, and HMGCR in hyperlipidemic mice fed a high-fat diet, thereby alleviating the associated inflammation as indicated by the reduction in pro-inflammatory cytokine levels [115].

Scientists at Merck developed siRNA oligonucleotide-lipid nanoparticles (siRNA-LNPs) targeting SCAP and demonstrated that this approach is effective in reducing hepatic SCAP mRNA expression [119]. As a result, hyperlipidemia is attenuated in rhesus monkeys and mice [119, 120], in the latter accompanied by decreased hepatic Ldlr and proprotein convertase subtilisin kexin/type 9 (Pcsk9) expression, repression of Srebp-regulated genes, and inhibition of *de novo* lipogenesis [119]. The serine kinase PCSK9, which is a therapeutic target for lipid-lowering drugs, is secreted by hepatocytes and subjects LDL receptors to lysosomal degradation [375].

The diarylthiazole fatostatin interacts with SCAP, inhibits its glycosylation, and blocks the transport of SREBP-1 from the ER to the Golgi, thereby suppressing SREBP-1 maturation [117, 376]. Fatostatin attenuates hepatic steatosis in obese mice while reducing body weight and blood glucose levels [117] and exhibits anti-tumoral/growth-retarding [377–379] and ferroptosis-inducing activity [379], which has been ascribed to impaired SREBP-1 activation [376]. We expect that fatostatin may also be cytotoxic to HCC cells, although this has not been explicitly demonstrated.

The pentacyclic lupane-type triterpenoid betulin from birch bark has a broad spectrum of pharmacological activities, among others, directed against metabolic disorders and cancer, including HCC [38, 66, 380]. Pleiotropic anti-tumoral mechanisms have been proposed for betulin, including inhibition of SREBP-1/2 maturation, which reduces fatty acid and cholesterol biosynthesis in HCC cells (2.3–100 μM betulin) and DEN-induced HCC in mice (gavage of 100 mg betulin/kg/day) [38, 66, 116]. Mechanistically, betulin physically interacts with SCAP (at 100 μM) and thereby promotes the interaction of SCAP with Insig1/2 to retain SREBPs at the ER [116]. Suppression of SREBP-1/2 and SREBP target genes in experimental murine HCC was accompanied by decreased mRNA expression of pro-inflammatory cytokines, such as TNFα [38]. While the authors confirm a functional link between SREBP processing and pro-inflammatory cytokine expression, the cytokine-lowering mechanism of betulin and its contribution to the anti-HCC activity remain diffuse, especially when considering that betulin may also target the TLR4 and nuclear factor (NF)-κB pathways [39].

Sulforaphane, an isothiocyanate from cruciferous vegetables, and the desaturated analog sulforaphene (30–100 μM, each) inhibit lipogenic enzyme expression in human Huh-7 hepatoma cells by promoting the ubiquitination and proteasomal degradation of pSREBP-1/2 in a SCAP-independent manner [122]. Central to the SREBP-1a-degrading activity of sulforaphane and sulforaphene is the SREPB-1a amino acid sequence from 595 to 784. The direct molecular target of sulforaphane responsible for the induction of SREBP-1/2 degradation remains elusive. Neither does sulforaphane interact directly with SREBPs, nor are known targets of sulforaphane, i.e., Kelch-like ECH-associated protein 1/nuclear factor erythroid 2-related factor 2 (KEAP1/NRF2) and heat shock protein 27 (HSP27), involved in pSREBP degradation [122]. Another study proposed that sulforaphane (1–20 μM; 5–20 mg/kg/day, p.o.) reduces hepatic lipogenic gene expression in rats on a high-fat diet by repressing the ER stress sensor protein kinase-like ER kinase (PERK) and decreasing SREPB-1 expression [123].

The bufadienolide cinobufotalin (0.1–0.4 μM) from the skin secretion of the giant toad inhibits both SREBP-1 expression and the binding of SREBP-1 to SREs in HepG2 cells, seemingly by interacting directly with the transcription factor, which together markedly represses the expression of lipogenic enzymes [61]. Cinobufotalin (2.5–5 mg/kg, i.p.) is effective *in vivo* and reduces lipogenesis and HCC tumor growth in grafted mice, the latter likely by inducing G2/M cell cycle arrest and apoptosis [61]. A meta-analysis of 27 clinical trials involving 2079 advanced HCC patients indicates that the combination of hepatic arterial chemoembolization (TACE) with adjuvant cinobufotalin injection is safe and more effective than TACE alone for the treatment of end-stage HCC [381].

Diverse small molecules and complex mixtures have been shown to decrease SREBP-1 expression or maturation in HCC cell lines, though the mode of action has often remained elusive (Table 1). These include pyridine co-ligand functionalized Pt(II) complexes [190]; the retinoic acid receptor β2 agonists AC261066 and AC55649 [176]; GPR40 agonists, such as GW9508, AMG-1638, and docosahexaenoic acid [179, 180]; and ethanolic extracts of several herbs, such as Zhiheshouwu (Polygoni multiflori Radix Praeparata) and Liriope platyphylla root [382, 383].

## Conclusion and perspective

Malignant transformation to HCC induces SREBP-1 signaling through a broad spectrum of regulatory mechanisms that enhance SREBP-1 expression, maturation, protein stability, and nuclear activity. This metabolic reprogramming confers advantages in survival, growth, proliferation, and dissemination to HCC, but also renders tumors sensitive to anti-lipogenic treatment. While the mechanistic insights into SREBP-1 regulation and function are rapidly increasing, pharmacological strategies that selectively target SREBP-1 signaling are still in their infancy. The main reasons for this are the limited availability of high-throughput screening assays for SREBP-1-interacting small molecules, the lack of obvious ligand binding pockets, and incomplete structural information. Crystal structures of SREBP-1 that could aid in the rational design of respective ligands have been solved, but are limited to the yeast ortholog and the N-terminal bHLH-Zip domain of the human transcription factor [384–386].

The vast majority of agents targeting SREBP-1 signaling are non-selective, either because of polypharmacological activities or because the upstream targets regulate multiple signaling pathways in addition to SREBP-1 activation/induction. Furthermore, many pathways, including kinase cascades (e.g., PI3K-Akt, mTORC1, AMPK) and transcription factors/co-activators (e.g., LXR), indirectly regulate SREBP-1 signaling. Because these pathways regulate multiple other cellular processes besides SREBP-1 that are involved in tumorigenesis, it is difficult to assess from genetic manipulation studies the extent to which the interference with SREBP-1 contributes to HCC suppression. Thus, many of the agents listed in Table 1 have been evaluated in pre-clinical studies and some in clinical trials, with the effective doses (in mg/kg body weight) used in (pre-)clinical studies reported whenever available. For example, the pan-kinase inhibitor sorafenib is a first-line treatment for HCC [387, 388], and the red wine stilbene resveratrol has been the subject of numerous clinical and epidemiological studies and meta-analyses (including a phase I trial in patients with liver metastases) [389]. AMPK activators have also been intensively studied in recent years for the treatment of metabolic diseases, including cancer, and several compounds have entered clinical evaluation [353]. While these compounds have been reported to interfere with SREBP-1 signaling and it is likely that this effect contributes to their overall efficacy, clinical and pre-clinical data do not allow conclusions as to whether the interference with SREBP-1 signaling mediates the observed beneficial and adverse effects.

Results from pharmacological approaches are even more difficult to interpret when considering that the vast majority of SREBP-1 modulators have been shown to have off-target effects; many other compounds have not been adequately studied. Available selective approaches to inhibit SREBP-1 signaling are largely limited to (i) the interaction of SREBP-1 and the upstream kinase PKM2 via peptide ligands (P8) [181], (ii) an apparent direct interaction of cinobufotalin with SREBP-1 (although little is known about potential other targets for this traditional Chinese medicine) [61], and (iii) oligonucleotide-based approaches via siRNA or decoy oligonucleotides [115, 119, 120]. Biopharmaceuticals such as therapeutic antibodies or SREBP-1-interacting proteins, which are expected to provide superior SREBP-1 selectivity, have not been explored so far. Of these more selective approaches, only cinobufotalin has been tested and shown to be effective in an in vivo HCC model [61] and clinical trials [381], while oligonucleotide approaches have been studied in hyperlipidemic animals (where they reduced lipogenesis and hepatic LDL uptake) [119, 120], and knowledge of P8 is limited to cell-based studies [181]. Such strategies may have the potential to achieve selectivity for SREBP-1 targeting. Other promising strategies to narrow down putative side effects (which we have not or only partially addressed here) include (i) selective inhibition of SREBP-1 target genes, such as ACC, FASN, SCD1, and ACLY [32, 390–392]; (ii) interference with defined, context-specific SREBP-1 regulators, e.g., PKD3 or CRTC2 [128, 198, 204]; (iii) functionalized nanoparticles for HCC targeting [393–395]; and (iv) tumor-specific gene therapy [223, 396].

Based on the above, the (still incompletely pharmacologically characterized) cinobufotalin seems to be currently at the forefront of agents that target SREBP-1 signaling with some selectivity. Nanoparticle-based approaches that deliver SREBP-1-interacting peptides or siRNA to target SREBP-1, SCAP, or other signaling molecules, as well as (not yet explored) therapeutic antibodies directed against these factors, hold great promise for the future. On the other hand, it is questionable whether selectivity is actually desirable to achieve efficacy against a complex disease such as HCC. Therefore, polypharmacological approaches based on the rational inhibition of SREBP-1, as already realized in several drugs and drug candidates, may pave the way to an improved clinical efficacy of rationally designed anti-cancer drugs, particularly in the treatment of HCC.

In addition, interesting new links between SREBP-1 and resistance to therapy (including chemo- and radioresistance) are emerging, driven in part by multiomics approaches [397–400], but the number of such studies exploring the role of SREBP-1 in therapy-resistant HCC is still very limited [27, 37, 44, 66, 71–74], and further research in this area is urgently needed. In this context, the bivalent role of SREBP-1 in conveying resistance in cancer (HCC) should be mentioned. High SREBP-1 levels increase HCC aggressiveness and resistance to classical chemotherapeutics, while sensitizing tumors to anti-lipogenic strategies and alternative forms of programmed cell death, such as ferroptosis [27, 42, 74, 98]*.*

In summary, selective SREBP-1 inhibitors are in high demand to investigate the pleiotropic, context-dependent functions of SREBP-1. Current anti-HCC strategies are instead dominated by multitarget small molecules that exhibit (subordinate) SREBP-1 inhibition, broadly interfere with lipogenesis, and may target additional cancer-promoting pathways. Whether they are inferior or superior towards monopharmacological approaches in terms of efficacy and safety remains to be determined in future studies.

## Data Availability

This review article does not contain original data but summarizes and discusses published data.

## Declarations

### Disclaimer

For the purpose of open access, the authors have applied a CC BY public copyright licence to any author accepted manuscript version arising from this submission.
